# Surface charge method for molecular surfaces with curved areal elements I. Spherical triangles

**DOI:** 10.1088/1361-648X/aaab2b

**Published:** 2018-03-14

**Authors:** Yi-Kuo Yu

**Affiliations:** National Center for Biotechnology Information, National Library of Medicine, National Institutes of Health, Bethesda, MD 20894, United States of America

**Keywords:** electrostatics, surface charge method, surface triangulation

## Abstract

Parametrizing a curved surface with flat triangles in electrostatics problems creates a diverging electric field. One way to avoid this is to have curved areal elements. However, charge density integration over curved patches appears difficult. This paper, dealing with spherical triangles, is the first in a series aiming to solve this problem. Here, we lay the ground work for employing curved patches for applying the surface charge method to electrostatics. We show analytically how one may control the accuracy by expanding in powers of the the arc length (multiplied by the curvature). To accommodate not extremely small curved areal elements, we have provided enough details to include higher order corrections that are needed for better accuracy when slightly larger surface elements are used.

## 1. Introduction

Not only of general importance at the molecular level, electrostatic interactions are crucial for biomolecular systems which consist of many charged molecules embedded in the polar solvent water. It is, however, impractical to apply precise quantum methods to even a moderately large biomolecular system. Therefore, numerous efforts were invested in classical approaches [1], which are often categorized according to how the solvent water is treated. Explicit solvent methods treat each water molecule at the level of atomic detail, while implicit solvent methods replace the individual water molecules with some type of smoothed out version.

Explicit solvent methods, such as TIPnP [2], allow description of biomolecular systems at finer detail, but their lack of incorporating mutual polarization of molecules can be a critical deficiency sometimes. The implicit solvent methods [3, 4] are in principle less computationally intensive when larger systems are considered; however, their application is limited to systems where fine details of solute-solvent interactions do not play a major role.

We have earlier suggested a classical model of dielectric spheres [5] and used it to approximate atoms and molecules by dielectric polarizable bodies [6]. If the bodies are embedded in a dielectric medium, this model becomes an implicit solvent model and the bodies represent the biomolecules while the solvent is modeled by the dielectric continuum. If, however, the bodies are considered to be in the vacuum, the model becomes an explicit solvent model in which biomolecules and waters are modeled explicitly, with their own parameters. This model readily includes polarization effect and was previously used to investigate the accuracy of our classical formalism in describing interactions between atomic-sized objects [6]. We find that the dielectric spheres model is surprisingly accurate down to distances where chemical bonds start to form. The formalism introduced in [5] allows one to find a solution with arbitrary accuracy for an arbitrary number of interacting dielectric spheres with point charges at their centers.

To extend this formalism to model molecules, one need to account for the non-spherical nature of chemical bonds by either introducing permanent and inducible higher multipoles [7] or expanding the scope of dielectric bodies to include non-spherical shapes. The former extension [7] is appropriate when the separation distances among the molecules are large and details of geometry play a lesser role; the latter extension may better represent regions of significant electronic density and thus might be suitable for shorter separations. For the latter, it is necessary to define the surface of the non-spherical molecule. A widely employed strategy for doing this is to create a grid of points at the outer atoms of the molecule. These points are then used to generate a set of flat triangular patches. The assumption is that one need only use a sufficient number of patches and then an accurate calculation will emerge. Indeed, the approximate surface certainly gets closer and closer to a smooth surface corresponding to the shape of the molecule. However, it does so at the expense of introducing more and more vertices, where the approximate surface has no well-defined normal direction.

For the purpose of finding the solvent accessible surface area or similar quantities, these points of undefined surface normal pose no problem (even though using curved surface element were shown to improve the results [8]). However, it is well known that at such points the electric field diverges. Therefore, when calculating the electrostatic interaction, the vertices of the flat patches create inherently uncontrollable sources of inaccuracy. Developing a better strategy for representing surfaces that are not completely spherical is thus a necessary task.

Two primary difficulties exist. First, with a constraint that the patch must include three specified spatial points, there exists no unique solution when curved surfaces are allowed. The parametrization of the patch becomes a problem. Second, even if one can parametrize a surface patch, one still needs a small expansion parameter to control the accuracy of the results obtained. In the dielectric sphere case, the small expansion parameter is naturally the radius of the sphere divided by the separation distances among the spheres. To overcome the first problem, we need a crisp rule for defining the molecular surface. For this, we use the molecular surface definition of Richards [9] and Connolly [10]. With this definition, only spherical (whole or partial) and partial toroidal surfaces come into play. When limited to those surfaces, however, a small intra-molecular expansion parameter emerges. It is the arc length on the surface multiplied by the local curvature.

This paper, focusing on spherical triangles, is the first in a series to investigate the use of curved surface elements for applying the surface charge method (SCM) [5, 11] in electrostatics. When a set of points on a sphere is chosen, the set of corresponding charge density values, one for each point/vertex, becomes the set of variables to be solved for. The basic idea of SCM is to write the electric potential and field near the dielectric interfaces in terms of the surface charge density values and then determine those values by applying the appropriate boundary condition.

In the sections below, we begin by describing the surface types that we shall encounter, followed by mentioning the fundamental equations needed for specifying a spherical triangle. We then describe, given the (not yet known) density values at the selected vertices, how may one smooth out the charge distribution completely independent of the coordinate choices. After illustrating explicitly the electric field discontinuity due to nonzero surface charge density, we proceed to calculate the electric field contribution right above/below a vertex of a spherical triangle from the surface charge density of that spherical triangle. We show there the emergence of a small expansion parameter. We then calculate the electric field produced by the charge density of a spherical triangle at a vertex of a non-neighboring spherical triangle. Here we also mention cases when numeric integration is the only way to proceed. We then consider, at a location outside a sphere *i*, the potential and the field contribution due to the charge density of a triangle on the sphere *i*. Some relevant mathematical formulas/derivations are provided in the appendices.

## 2. Surface types

In the context of implicit solvent, one may use a probe sphere to represent the solvent molecule and represent each atom by a sphere of finite radius. This leads to two surfaces: the solvent accessible surface and the molecular surface. The former is defined by the surface traced by the center of the probe sphere while rolling around the atoms of a molecule; the latter, the molecular surface [9, 10, 12], is defined by tracing the inwardfacing surface of the probe. In the context of explicit solvent model, molecules of interest and solvent molecules are modeled separately. Here a probe sphere is introduced simply to smooth out the surfaces of dielectric objects so that the surface normal is well defined everywhere in order to properly apply the boundary condition; the size of the probe has no direct physical interpretation.

When using the molecular surface definition [9, 10, 12] above, there will be three types of surface regions for each generic molecular surface. The type I surface are inner toroidal surfaces, made of the inward-facing surface of the probe, that occur when rolling the probe sphere between two atoms. The type II are the partial inward-facing surfaces of the probe sphere right at the stopping point while its rolling between two atoms is terminated by other atoms in the way. The type III are partial outward-facing spherical surfaces associated with different atoms. It is important to note that the adjoining partial surfaces can only be either the jointing of type I and type II or the jointing of type I and type III. Type II and type III surfaces do not join together. Furthermore, the jointing line between different surface types, when viewed from the perspective of the associated spheres, can be parameterized in such a way that it is of constant polar angle (i.e. only the azimuthal angle varies).

When treating a molecule as an dielectric object with a prescribed dielectric constant and surface delineated by the method of Richards [9] and Connolly [10, 12], we know from basic electrostatics that the induced charge only exists on the dielectric boundaries. Consequently, in terms of numerically solving the associated electrostatic problem, we need to assign a charge density value for each surface elements on the dielectric boundary. As we mentioned in the Introduction, using flat triangulation will lead to the undesirable consequence of a diverging electric field. Our aim here is to replace the flat triangles with other curved surface elements to eliminate the unphysical diverging electric field. For surface elements, we choose to use spherical triangles for type II and type III partial surfaces and curved rectangles for type I partial surfaces. This last point will be elaborated later in a subsequent manuscript.

One important point to note is that the curves connecting surface type I and surface type III (or II) can be viewed as a curve of fixed latitude on surface type III (or II). This introduces a special need for treating the surface element as we will detail in the subsequent manuscript. Our main objective is to calculate the electric field produced by the curved surface elements to minimize the amount of brute force numerical integration. In order to have a smooth surface charge density profile, it is also important to develop a method to interpolate the charge density values from the charge densities specified at discrete points. We shall show how we may achieve this goal in a coordinate free fashion.

## 3. Spherical triangles

When we partition (a part of) a spherical surface, we may first choose individual points, and then connect between points with the big arc. This uniquely specifies a spherical triangle when given its three vertices. For two points along the same longitude, the great arc is simply the segment of the longitude line connecting these two points; the great arc connecting two points of different longitude is given by the geodesic equation:

(1) $$\cot\;\theta =a\;\cos(\phi -\phi_{0}).$$

(For completeness, a derivation is provided in appendix A.)

### 3.1. Parameters for geodesic equation

Without losing generality, let us denote the first of the three vertices of the spherical triangle as the north pole of the sphere. Let the second vertex have spherical coordinates (*θ* = *β*_3_, *ϕ* = 0) and the third vertex have coordinates (*θ* = *β*_2_, *ϕ* = *α*). Obviously, the great arcs connecting the north pole to both (*β*_3_, 0) and (*β*_2_, *α*) are longitudinal lines. The reason we let the second vertex have spherical coordinates (*β*_3_, 0) is because *β*_3_ is the length of the arc opposite to vertex 3 (if the sphere has radius 1). The only nontrivial arc is the one connecting (*β*_3_, 0) and (*β*_2_, *α*) However, this line must be describable by equation (1), and by equating the values

(2) $$\cot\;\beta_{3}=a\;\cos(-\phi_{0})=a\;\cos(\phi_{0})$$

(3) $$\cot\;\beta_{2}=a\;\cos(\alpha -\phi_{0})=a(\cos\;\phi_{0}\;\cos\;\alpha +\sin\;\phi_{0}\;\sin\;\alpha )$$

we may learn the parameters of the geodesic. Note that we must have 0 < *α* < *π*. We then express *ϕ*_0_ and *a* in terms of the angle *α*. Multiplying equation (2) by cos *α* and subtracting it from both sides of equation (3) we obtain

$$\cot\;\beta_{2}-\cos\;\alpha \;\cot\;\beta_{3}=a\;\sin\;\alpha \;\phi_{0}.$$

Multiplying both sides of equation (2) by sin *α*, we have

$$\sin\;\alpha \;\cot\;\beta_{3}=a\;\sin\;\alpha \;\cos\;\phi_{0}.$$

We thus have (using the ratio of the two equations above)

(4) $$\tan\;\phi_{0}=\frac{\tan\;\beta_{3}-\cos\;\alpha \;\tan\;\beta_{2}}{\sin\;\alpha \;\tan\;\beta_{2}}=\frac{\frac{\tan\;\beta_{3}}{\tan\;\beta_{2}}-\cos\;\alpha }{\sin\;\alpha }.$$

With similar algebraic operations, we also have

(5) $$\tan(\alpha -\phi_{0})=\frac{\frac{\tan\;\beta_{2}}{\tan\;\beta_{3}}-\cos\;\alpha }{\sin\;\alpha }.$$

From equation (4), we also have

(6) $$\phi_{0}=\tan^{-1}\left(\frac{\tan\;\beta_{3}-\cos\;\alpha \;\tan\;\beta_{2}}{\sin\;\alpha \;\tan\;\beta_{2}}\right).$$

Using $\cos\;\phi_{0}=\sqrt{1∕(1+\tan^{2}\;\phi_{0})}$, we have

$$\cos\;\phi_{0}=\frac{\sin\;\alpha \;\tan\;\beta_{2}}{\sqrt{{\left(\tan\;\beta_{3}-\cos\;\alpha \;\tan\;\beta_{2}\right)}^{2}+\sin^{2}\;\alpha \;\tan^{2}\beta_{2}}},$$

and with equation (2), we obtain

(7) $$a=\frac{\cot\;\beta_{3}}{\cos\;\phi_{0}}=\frac{\sqrt{\cot^{2}\beta_{2}+\cot^{2}\beta_{3}-2\;\cos\;\alpha \;\cot\;\beta_{2}\;\cot\;\beta_{3}}}{\sin\;\alpha }.$$

One interesting exercise is to find the length of the great arc connecting point 2 and 3. Given equation (1), we know

$$ds=\sqrt{\sin^{2}\theta +{\left(\frac{d\theta }{d\phi }\right)}^{2}}d\phi =\sqrt{\frac{1}{1+a^{2}\cos^{2}(\phi -\phi_{0})}+\frac{a^{2}\sin^{2}(\phi -\phi_{0})}{{\left[1+a^{2}\cos^{2}(\phi -\phi_{0})\right]}^{2}}}d\phi =\frac{\sqrt{1+a^{2}}}{1+a^{2}\cos^{2}-(\phi -\phi_{0})}d\phi .$$

And the great arc angle is thus given by

(8) $$\beta_{1}=\int_{0}^{\alpha }\frac{\sqrt{1+a^{2}}}{1+a^{2}\cos^{2}(\phi -\phi_{0})}d\phi =\tan^{-1}\frac{\tan(\alpha -\phi_{0})}{\sqrt{1+a^{2}}}+\tan^{-1}\frac{\tan(\phi_{0})}{\sqrt{1+a^{2}}}.$$

Using equation (7), we have

$$a^{2}\sin^{2}\alpha =\cot\;\beta_{2}\;\cot\;\beta_{3}\left[\frac{\tan\;\beta_{2}}{\tan\;\beta_{3}}+\frac{\tan\;\beta_{3}}{\tan\;\beta_{2}}-2\;\cos\;\alpha \right]$$

and

$$\begin{array}{cc} (1+a^{2})\; & \sin^{2}\;\alpha =\sin^{2}\alpha +\cot^{2}\beta_{2}+\cot^{2}\beta_{3}-2\;\cos\;\alpha \;\cot\;\beta_{2}\;\cot\;\beta_{3}\\ = & 1-\cos^{2}\alpha +\cot^{2}\beta_{2}+\cot^{2}\beta_{3}+\cot^{2}\;\beta_{2}\;\cot^{2}\beta_{3}\\ -\; & 2\;\cos\;\alpha \;\cot\;\beta_{2}\;\cot\;\beta_{3}-\cot^{2}\beta_{2}\;\cot^{2}\;\beta_{3}\\ = & (1+\cot^{2}\beta_{2})(1+\cot^{2}\beta_{3})-{\left(\cos\;\alpha +\cot\;\beta_{2}\;\cot\;\beta_{3}\right)}^{2}\\ = & \frac{1-{\left({\hat{r}}_{2}\cdot {\hat{r}}_{3}\right)}^{2}}{\sin^{2}\beta_{2}\sin^{2}\beta_{3}}=\frac{\sin^{2}\beta_{1}}{\sin^{2}\beta_{2}\sin^{2}\beta_{3}}. \end{array}$$

Hence

(9) $$\sqrt{1+a^{2}}=\frac{\sin\;\beta_{1}∕\sin\;\alpha }{\sin\;\beta_{2}\;\sin\;\beta_{3}}.$$

Equations (8) and (9) will be useful later.

When *β*_2_ = *β*_3_ = *β*, we have from equation (6)

(10) $$\phi_{0}=\tan^{-1}\left(\frac{1-\cos\;\alpha }{\sin\;\alpha }\right)=\tan^{-1}\left(\tan\frac{\alpha }{2}\right)=\frac{\alpha }{2},$$

and $a=\frac{\cot\;\beta }{\cos\;\frac{\alpha }{2}}$ is obviously of order 1/*β* when the *β* angles are small. When *β*_2_ ≠ *β*_3_, equation (4) implies that $-\frac{\cos\;\alpha }{\sin\;\alpha }<\;\tan\;\phi_{0}<\frac{\tan\;\beta_{3}∕\tan\;\beta_{2}}{\sin\;\alpha }$, or $\tan(\alpha -\pi ∕2)<\tan\;\phi_{0}<\frac{\tan\;\beta_{3}∕\tan\;\beta_{2}}{\sin\;\alpha }$, implying

$$\alpha -\pi ∕2<\phi_{0}<\pi ∕2-\tan^{-1}\left(\frac{\sin\;\alpha \;\tan\;\beta_{2}}{\tan\;\beta_{3}}\right),$$

or

$$-\pi ∕2+\tan^{-1}\left(\frac{\sin\;\alpha \;\tan\;\beta_{2}}{\tan\;\beta_{3}}\right)<-\phi_{0}<-\alpha +\pi ∕2.$$

These inequalities ensure that when 0 ⩽ *ϕ* ⩽ *α*, both cos(*ϕ*_0_) and cos(*ϕ* − *ϕ*_0_) are positive.

### 3.2. Smooth charge density parametrization

On the three vertices of a spherical triangle, one may assign three charge density values. Physically speaking, the charge distribution should be smooth and thus we seek to interpolate the charge density values from those specified on the three vertices. Along a great arc side connecting two vertices of the spherical triangle, we expect the charge density to vary smoothly along the side from one vertex to the other. The charge density profile also should be smooth within each spherical triangle. To achieve this goal, we adapt the following interpolation scheme starting with a smooth charge distribution on a flat triangle and project it to the spherical triangle.

**Figure 1.**
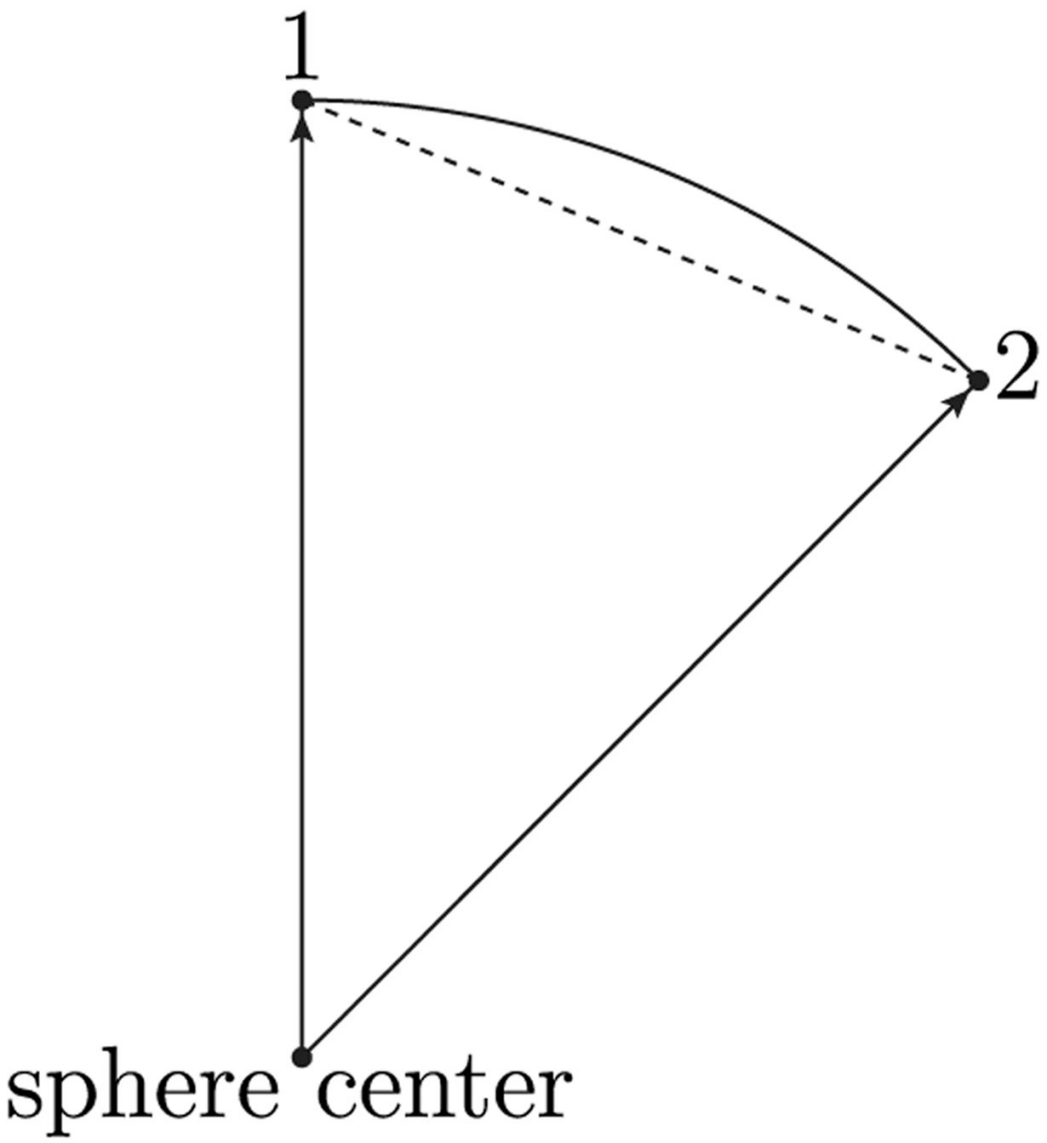
The slice containing center of the sphere and vertices 1 and 2. The dashed line connecting vertices 1 and 2 is also an edge of the underlying flat triangle. Evidently, any point on the dashed line, when extending the vector from the sphere center to that point will reach a corresponding point on the great arc $\overset{⌢}{12}$.

Picking any two of the three vertices of the sphereical triangle, one finds that the vectors pointing from the sphere center to these two vertices form a plane that contains the great arc connecting these two vertices. For example, let us focus on the plane containing the sphere center, vertex 1 (the north pole), and vertex 2 (the point with coordinates (*θ* = *β*_3_, *ϕ* = 0)). Vectors from the center of the sphere to any point on the line can be extended to the surface of the sphere, the collection of which forms the great arc connecting vertex 1 and vertex 2 on the spherical surface, see figure 1 as an illustration. Any point in the interior of the flat triangle is thus mapped to a point inside the spherical triangle via extending vectors originating from the sphere center to points on the flat triangle further outward to the sphere surface. Evidently, this mapping is one to one and continuous, meaning that close points on the flat triangle are mapped to close points on the sphere.

**Figure 2.**
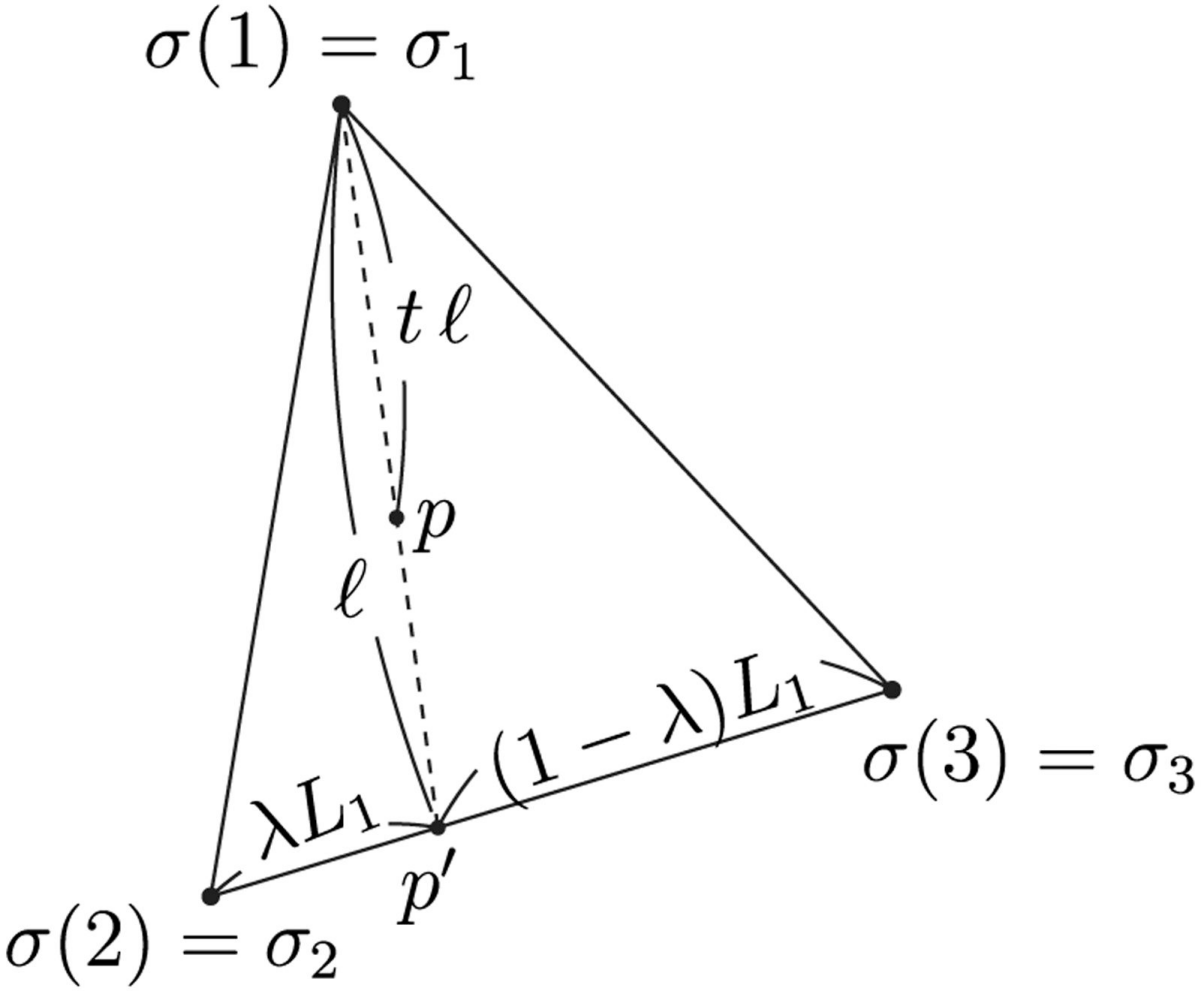
The flat triangle containing the three vertices 1–3 of the spherical triangle. The charge density values are first assigned to the flat triangle and then extended to the spherical triangle. The charge density values of a point p on the flat triangle is given by (1 − *t*) *σ*_1_ + *t* [(1 − *λ*)*σ*_2_ + *λσ*_3_].

We next build a smooth function on the flat triangle and then extend it to the spherical triangle to achieve a continuous charge distribution over all spherical triangles. Assume that we assign a density value *σ*_*i*_ to vertex *i*. A simple way to build a smooth function is to interpolate linearly on the flat triangle. For any point *p* inside the flat triangle, let us extend the line from vertex 1 to *p* further till it reaches the edge *L*_1_ connecting vertices 2 and 3, and let us call the intersection point *p*′. See figure 2 for a graphical illustration. Let the line length from vertex 1 to *p*′ be *ℓ* and the line length from vertex 1 to *p* be *tℓ* (with 0 ⩽ *t* ⩽ 1). Let us also use *L*_1_ to represent the length of edge *L*_1_. Now assume the distance between vertex 2 and *p*′ to be *L*_1_ *λ* (with 0 ⩽ *λ* ⩽ 1). It is natural to assign the charge density (1 − *λ*) *σ*_2_ + *λσ*_3_ to point *p*′. Similarly, for point *p*, we may assign the charge density to be (1 − *t*) *σ*_1_ + *t* [(1 − *λ*)*σ*_2_ + *λσ*_3_] = *σ*_1_ + *t* [(1 − *λ*)*σ*_2_ + *λσ*_3_ − *σ*_⋅1_] Note that at the center of mass of the flat triangle where *λ* = 1/2 and *t* = 2/3, we find that the interpolated density takes the desirable value (*σ*_1_ + *σ*_2_ + *σ*_3_)/3.

**Figure 3.**
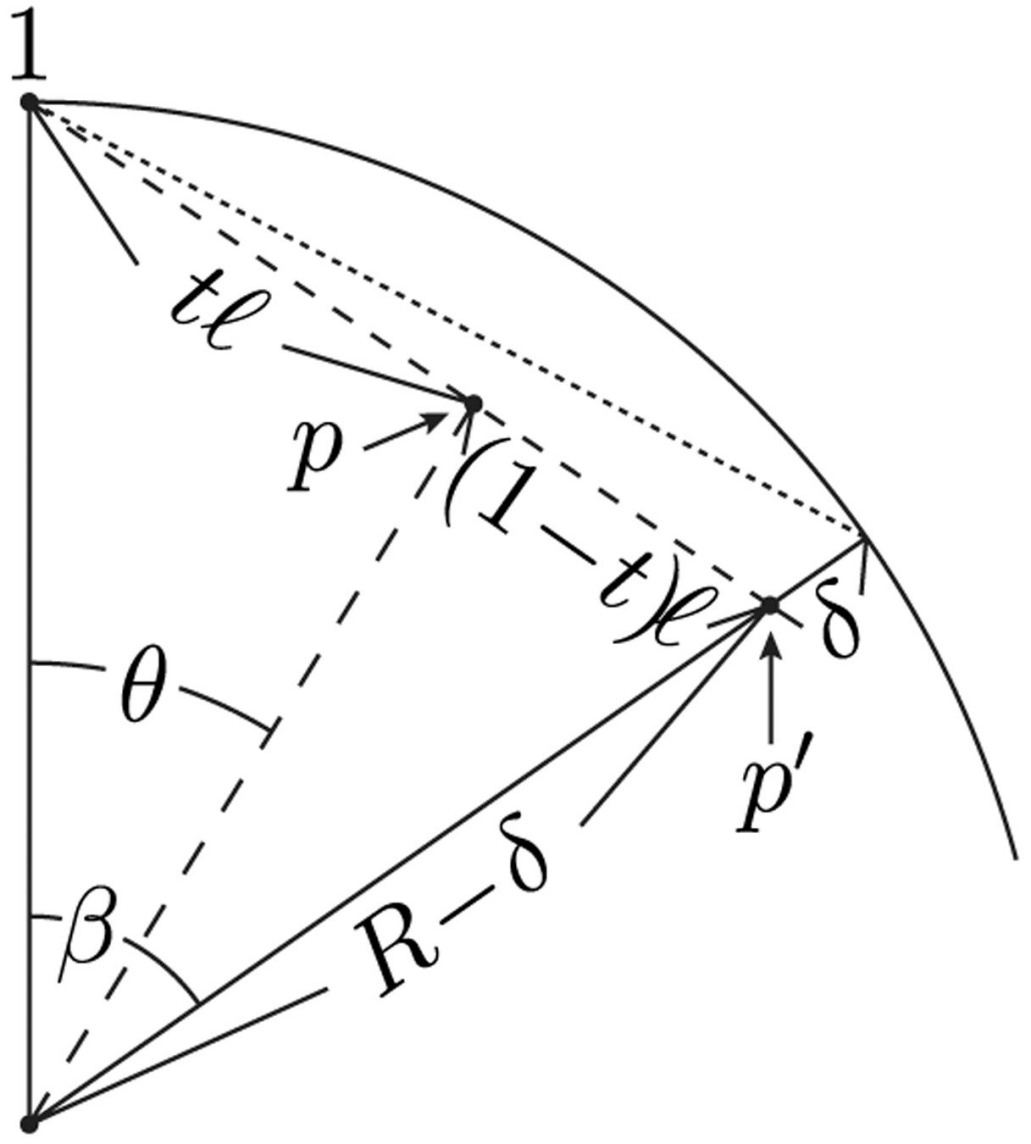
The constant longitude slice through center of the sphere, vertex 1, and point *p*′ that lies on the straight line segment connecting vertices 1 and 2.

Under this parametrization, we may view *λ* as dependent on the azimuthal angle, and for any given azimuthal angle (or specified *λ*) the parameter *t* evidently depends on the polar angle *θ*. Picking an azimuthal angle 0 ⩽ *ϕ* ⩽ *α*, a corresponding boundary polar angle *β*(*ϕ*) is obtained by using equations (1), (6), and (7). Let us now look at the spherical slice through vertex 1 at azimuthal angle *ϕ*, see figure 3. Without loss of generality, let us view this slice as the *x* — *z* plane. The vector from vertex 1 to *p*′ equals

$$\begin{array}{cc} & \left((R-\delta )\sin\;\beta ,0,(R-\delta )\cos\;\beta )-(0,0,R)=((R-\delta \right)\\ & \sin\;\beta ,0,(R-\delta )\cos\;\beta -R) \end{array}$$

and the coordinates of *p* become

$$\begin{array}{cc} & \left(0,0,R)+t((R-\delta )\;\sin\;\beta ,0,(R-\delta )\;\cos\;\beta -R\right)\\ & =(t(R-\delta )\;\sin\;\beta ,0,(1-t)R+t(R-\delta )\;\cos\;\beta ). \end{array}$$

The ratio of the *x*-component to the *z*-component of the expression above yields tan *θ*,

(11) $$\begin{array}{cc} \tan\;\theta = & \frac{t(R-\delta )\;\sin\;\beta }{(1-t)R+t(R-\delta )\;\cos\;\beta }\text{or}\\ t(\theta )= & \frac{R\;\tan\;\theta }{[R-(R-\delta )\;\cos\;\beta ]\;\tan\;\theta +(R-\delta )\;\sin\;\beta }\\ = & \frac{\sin\;\theta }{[1-(1-\delta ∕R)\;\cos\;\beta ]\;\sin\;\theta +[(1-\delta ∕R)\;\sin\;\beta ]\;\cos\;\theta }\\ \equiv & \frac{\sin\;\theta }{C(\phi )\;\sin\;\theta +D(\phi )\;\cos\;\theta }. \end{array}$$

Note that both *β* and *δ* depend on the azimuthal angle *ϕ*. We shall unearth their *ϕ* dependence next. We now return to the Cartesian coordinate system in which the coordinates of vertex 1 are **r**_1_ = (0, 0, *R*), vertex 2: **r**_2_ = (*R* sin *β*_3_, 0, *R* cos *β*_3_), and vertex 3: **r**_3_ = (*R* sin *β*_2_ cos *α*, *R* sin *β*_2_ sin *α*, *R* cos *β*_2_). The coordinates of *p*′ then read

$$\begin{array}{cc} r_{2}+\lambda (r_{3}-r_{2}) & =((1-\lambda )R\;\sin\;\beta_{3}+\lambda R\;\sin\;\beta_{2}\;\cos\;\alpha ,\\ \lambda R\;\sin & \;\beta_{2}\;\sin\;\alpha ,(1-\lambda )R\;\cos\;\beta_{3}+\lambda R\;\cos\;\beta_{2}). \end{array}$$

Evidently, the ratio of the *y*-component to the *x*-component yields tan *ϕ*,

(12) $$\begin{array}{cc} \tan\;\theta & =\frac{\lambda \;\sin\;\beta_{2}\;\sin\;\alpha }{(1-\lambda )\;\sin\;\beta_{3}+\lambda \;\sin\;\beta_{2}\;\cos\;\alpha }\;\;\text{or}\\ \lambda (\phi ) & =\frac{\sin\;\beta_{3}\;\tan\;\phi }{\sin\;\beta_{2}\;\sin\;\alpha +(\sin\;\beta_{3}-\sin\;\beta_{2}\;\cos\;\alpha )\;\tan\;\phi }\\ & =\frac{\sin\;\beta_{3}\;\sin\;\phi }{\sin\;\beta_{2}\;\sin(\alpha -\phi )+\sin\;\beta_{3}\;\sin\;\phi }. \end{array}$$

As for the quantity *δ*(*ϕ*), we note that it is defined as

(13) $$\begin{array}{cc} \delta (\phi ) & =R-|r_{2}+\lambda (r_{3}-r_{2})|=R-\sqrt{{r_{2}}^{2}+\lambda^{2}{\left(r_{3}-r_{2}\right)}^{2}+2\lambda r_{2}\cdot (r_{3}-r_{2})}\\ & =R\left[1-\sqrt{1+2\lambda^{2}(1-{\hat{r}}_{2}\cdot {\hat{r}}_{3})+2\lambda ({\hat{r}}_{2}\cdot {\hat{r}}_{3}-1)}\right]\\ & =R\left[1-\sqrt{1-2\lambda (1-\lambda )(1-{\hat{r}}_{2}\cdot {\hat{r}}_{3})}\right] \end{array}$$

where ${\hat{r}}_{2}=r_{2}∕R$ and ${\hat{r}}_{3}=r_{3}∕R$ are unit vectors along the directions of **r**_2_ and **r**_3_ respectively. Evidently, 0 ⩽ *λ* ⩽ 1, and the smallness of *δ*(*ϕ*)/*R* depends on how close ${\hat{r}}_{2}\cdot r_{3}=\sin\;\beta_{2}\;\sin\;\beta_{3}\;\cos\;\alpha +\cos\;\beta_{2}\;\cos\;\beta_{3}$ is to 1. For small *β*_2_ and *β*_3_, $\delta (\phi )∕R\approx \lambda (1-\lambda )(1-{\hat{r}}_{2}\cdot {\hat{r}}_{3})$ is $O(\beta^{2})$. Ultimately, the *ϕ* dependence of *δ* comes from *λ*(*ϕ*) in equation (12).

With all the needed components developed, we may now write down the interpolated charge density profile on the spherical triangle as

(14) $$\begin{array}{cc} \sigma (\theta ,\phi ) & =\sigma_{1}+t(\theta )[[1-\lambda (\phi )]\sigma_{2}+\lambda (\phi )\sigma_{3}-\sigma_{1}]\\ & =\sigma_{1}+t(\theta )[\sigma_{2}-\sigma_{1}+\lambda (\phi )(\sigma_{3}-\sigma_{2})]\\ & \equiv \sigma_{1}+t(\theta )B(\phi ), \end{array}$$

where *t*(*θ*) is given by equation (11) and *⩽*(*ϕ*) is given by equation (12). Note that for a given *ϕ* angle, *θ* ranges from 0 to *β*(*ϕ*), which by equation (1) is

(15) $$\beta (\phi )=\cot^{-1}(a\;\cos(\phi -\phi_{0}))$$

with *ϕ*_0_ given by equation (6) and *a* by equation (7).

Given the coordinate choice of the three vertices (with one being the north pole of the sphere) of the spherical triangle and the charge interpolation scheme above, we may now compute the electric field produced by the spherical triangle at any arbitrary point. We separate three cases: first, the potentials right above and below the north pole; second, the electric field at any point on the same sphere but outside the spherical triangle; third, the electric field at any point outside the sphere.

### 3.3. Isolating electric field discontinuity right above and below the north pole

For the first case mentioned above, the surface charge density right at the north pole produces electric field of opposite direction right above and below the north pole. This suggests that one may calculate directly the electric field right at the north pole and incorporate the discontinuity of electric field afterwards. To illustrate this idea, let us consider the electric field produced by a spherical cap (parametrized by 0 ⩽ *θ* ⩽ *β*, 0 ⩽ *ϕ* ⩽ 2*π*) with uniform surface charge density *σ*. In the Cartesian coordinate system, a point near the north pole has coordinates (0, 0, *R* + *ϵ*), with *ϵ* > 0 (*ϵ* < 0) indicating point right above (below) the north pole. Of course, the north pole location is at *ϵ* = 0.

Consider the electric field produced by the spherical cap. Due to azimuthal symmetry, the net field can only be along the $\hat{z}$ direction:

(16) $$\begin{array}{cc} E_{z}(R+\epsilon ) & =\int_{0}^{2\pi }d\phi \int_{0}^{\beta }\frac{d\theta (R+\epsilon -R\cos\;\theta )\sigma R^{2}\;\sin\;\theta }{{\left[{\left(R\;\sin\;\theta \;\cos\;\phi \right)}^{2}+{\left(R\;\sin\;\theta \;\sin\;\phi \right)}^{2}+{\left(R+\epsilon -R\;\cos\;\theta \right)}^{2}\right]}^{3∕2}}\\ & =\sigma R^{2}\int_{0}^{2\pi }d\phi \int_{0}^{\beta }\sin\;\theta d\theta \frac{R+\epsilon -R\;\cos\;\theta }{{\left[{\left(R+\epsilon \right)}^{2}+R^{2}-2R(R+\epsilon )\;\cos\;\theta \right]}^{3∕2}}\\ & =2\pi \sigma s^{2}\int_{0}^{\beta }\sin\;\theta d\theta \frac{1-s\;\cos\;\theta }{{\left[1+s^{2}-2s\;\cos\;\theta \right]}^{3∕2}} \end{array}$$

with *s* ≡ *R*/(*R* + *ϵ*). Since we are interested in the limit ∣*ϵ*∣ → 0, we consider the limit *s* → 1. When *s* → 1 from the *s* < 1 end, it means *ϵ* > 0, corresponding to the case when the point is right above the north pole. When *s* → 1 from *s* > 1 end, it means *ϵ* < 0, corresponding to the case when the point is right below the north pole. Continuing the calculation, we find

(17) $$\begin{array}{cc} E_{z}(R+\epsilon ) & =2\pi \sigma s^{2}\int_{\cos\;\beta }^{1}dx\frac{(1+s^{2})∕2-\mathrm{sx}+(1-s^{2})∕2}{{\left(1+s^{2}-2\mathrm{sx}\right)}^{3∕2}}\\ = & 2\pi \sigma s^{2}\int_{\cos\;\beta }^{1}dx\left[\frac{1}{2\sqrt{1+s^{2}-2\mathrm{sx}}}+\frac{1-s^{2}}{2{\left(1+s^{2}-2\mathrm{sx}\right)}^{3∕2}}\right]\\ = & 2\pi \sigma s^{2}\left[\frac{\sqrt{1+s^{2}-2s\;\cos\;\beta }-|1-s|}{2s}+\frac{1-s^{2}}{2s|1-s|}-\frac{1-s^{2}}{2s\sqrt{1+s^{2}-2s\;\cos\;\beta }}\right]\\ \Rightarrow & 2\pi \sigma \left[\sqrt{(1-\cos\;\beta )∕2}+\mathrm{sgn}(1-s)\right]\;\text{as}\;s\to 1. \end{array}$$

Note that when *ϵ* = 0, the integral (16) superficially reduces to $2\pi \sigma \sqrt{(1-\cos\;\beta )∕2}$, which is exactly the first contribution in the equation above. The second (nontrivial) contribution in the equation above arises from the difference between the electric fields right above and below the north pole. It contributes 2*πσ* (−2*πσ*) for the point immediately above (below) the north pole. This indicates that we may obtain the correct field strength by naïvely computing the field right at the north pole and add the discontinuous part afterwards.

### 3.4. Electric field near the north pole (a vertex of the charged spherical triangle)

Without loss of generality, we can rotate the coordinates such that the vertex of interest (near which the electric field should be calculated) becomes the north pole, and the other two vertices have coordinates (*θ* = *β*_3_, *ϕ* = 0) and (*θ* = *β*_2_, *ϕ* = *α*). Obviously, the great arcs connecting the north pole to both (*β*_3_, 0) and (*β*_2_, *α*) are longitudinal lines. The great-circle arc connecting (*β*_3_, 0) and (*β*_2_, *α*), on the other hand, follows the parametric form (1) with parameters specified by equations (6) and (7) in section 3.1.

Using the notation and development in section 3.2, we may write the continuous portion of the electric field along the $\hat{z}$ direction at the north pole (vertex 1) as

(18) $$\begin{array}{cc} E_{z} & =\int_{0}^{\alpha }d\phi \int_{0}^{\beta (\phi )}\frac{\sigma (\theta ,\phi )\;\sin\;\theta \;d\theta }{2\sqrt{2}\sqrt{1-\cos\;\theta }}\\ = & \int_{0}^{\alpha }d\phi \int_{0}^{\beta (\phi )}\frac{\sin\;\theta \;d\theta }{2\sqrt{2}}\frac{\sigma_{1}+t(\theta )[\sigma_{2}-\sigma_{1}+\lambda (\phi )(\sigma_{3}-\sigma_{2})]}{\sqrt{1-\cos\;\theta }} \end{array}$$

with *t*(*θ*) and *λ*(*ϕ*) given by equations (11) and (12) respectively. The *θ* integral in $\int_{0}^{\alpha }d\phi \int_{0}^{\beta (\phi )}\frac{\sigma_{1}\;\sin\;\theta \;d\theta }{2\sqrt{2}\sqrt{1-\cos\;\theta }}$ can be done easily

(19) $$\int_{0}^{\alpha }d\phi \frac{\sigma_{1}}{2\sqrt{2}}\int_{\cos\;\beta (\phi )}^{1}\frac{dx}{\sqrt{1-x}}=\sigma_{1}\int_{0}^{\alpha }d\phi {\left(\frac{1-\cos\;\beta (\phi )}{2}\right)}^{1∕2}.$$

When the *β*_2_ and *β*_3_ angles are small, the parameter *a* in equation (1) is large, implying that *β*(*ϕ*) is also small. Consequently, one may expand 1 − cos *β*(*ϕ*) using

(20) $$\begin{array}{cc} \cos\;\beta (\phi ) & =\frac{a\;\cos(\phi -\phi_{0})}{{\left(1+a^{2}\;\cos^{2}(\phi -\phi_{0})\right)}^{1∕2}}=\frac{1}{\sqrt{1+1∕[a^{2}\;\cos^{2}(\phi -\phi_{0})]}}\\ \approx 1 & -\frac{1}{2[a^{2}\;\cos^{2}(\phi -\phi_{0})]}+\frac{3}{8{\left[a^{2}\;\cos^{2}(\phi -\phi_{0})\right]}^{2}}-\frac{5}{16{\left[a^{2}\;\cos^{2}(\phi -\phi_{0})\right]}^{3}}+\cdots \end{array}$$

and

$${\left(\frac{1-\cos\;\beta (\phi )}{2}\right)}^{1∕2}\approx \frac{1}{2a\;\cos(\phi -\phi_{0})}\left(1-\frac{3∕8}{a^{2}\cos^{2}(\phi -\phi_{0})}+\frac{31∕128}{a^{4}\;\cos^{4}(\phi -\phi_{0})}+\cdots \right).$$

In fact, the above expansion can be evaluated to arbitrary accuracy since 1/*a* is of order *β* (see equation (7))and the denominator of every term contains odd powers of *a* cos(*ϕ* − *ϕ*_0_) whose integral can be calculated

(21) $$\begin{array}{cc} \int_{0}^{\alpha } & d\phi \frac{1}{\cos^{2\ell +1}(\phi -\phi_{0})}=\int_{-\phi_{0}}^{\alpha -\phi_{0}}\frac{d\;\sin\phi }{{\left[1-\sin^{2}\phi \right]}^{\ell +1}}=\int_{-\sin\;\phi_{0}}^{\sin(\alpha -\phi_{0})}\frac{dy}{{\left[1-y^{2}\right]}^{\ell +1}}\\ & ={\left[\frac{(2\ell -1)!!}{2^{\ell +1}\ell !}\text{ln}\frac{1+y}{1-y}+\sum_{p=0}^{\ell -1}\frac{(2\ell -1)!!(\ell -p-1)!}{2^{p+1}\ell !(2\ell -1-2p)!!}\frac{y}{{\left(1-y^{2}\right)}^{\ell -p}}\right]}_{-\sin\;\phi_{0}}^{\sin(\alpha -\phi_{0})} \end{array}$$

with the understanding that both (−1)!! and 0! are defined to be 1.

We now turn to the integral containing *t*(*θ*) = sin *θ*/[*C*(*ϕ*) sin *θ* + *D*(*ϕ*) cos *θ*], with *D*(*ϕ*) = (1 − *δ*/*R*) sin *β* and *C*(*ϕ*) = (1 − cos *β*) + (*δ*/*R*) cos *β*. Note that *D*(*ϕ*) is O(β) while *C*(*ϕ*) is $O(\beta^{2})$ because both 1 − cos *β* and *δ*/*R* are $O(\beta^{2})$. The *θ* integral in equation (18) can now be evaluated:

(22) $$\begin{array}{cc} \int_{0}^{\beta (\phi )}\frac{\sin\;\theta \;d\theta }{2\sqrt{2}}\frac{t(\theta )}{\sqrt{1-\cos\;\theta }} & =\frac{1}{2\sqrt{2}}\int_{\cos\;\beta (\phi )}^{1}\frac{dx}{\sqrt{1-x}}\frac{\sqrt{1-x^{2}}}{C\sqrt{1-x^{2}}+\mathrm{Dx}}\\ =\frac{1}{2\sqrt{2}}\int_{\cos\;\beta (\phi )}^{1} & \frac{dx}{\sqrt{1-x}}\frac{\sqrt{1-x^{2}}[C\sqrt{1-x^{2}}-\mathrm{Dx}]}{\left[C\sqrt{1-x^{2}}+\mathrm{Dx}][C\sqrt{1-x^{2}}-\mathrm{Dx}\right]}\\ =\frac{1}{2\sqrt{2}}\int_{\cos\;\beta (\phi )}^{1} & \frac{dx}{\sqrt{1-x}}\left[\frac{C(1-x^{2})}{C^{2}-(C^{2}+D^{2})x^{2}}-\frac{\mathrm{Dx}\sqrt{1-x^{2}}}{C^{2}-(C^{2}+D^{2})x^{2}}\right]. \end{array}$$

The first part of integral (22) can be rewritten as (with *η*^2^ ≡ (*C*^2^ + *D*^2^)/*C*^2^)

(23) $$\begin{array}{cc} & \frac{1}{2\sqrt{2}\eta^{2}C}\int_{\cos\;\beta (\phi )}^{1}\frac{dx}{\sqrt{1-x}}\frac{\eta^{2}x^{2}-1+(1-\eta^{2})}{\eta^{2}x^{2}-1}\\ & \;\;=\frac{1}{2\sqrt{2}\eta^{2}C}\int_{\cos\;\beta (\phi )}^{1}\frac{dx}{\sqrt{1-x}}\left[1-\frac{\eta^{2}-1}{2}\left(\frac{1}{\eta x-1}-\frac{1}{\eta x+1}\right)\right]\\ & \;\;=\frac{1}{2\sqrt{2}\eta^{2}C}\left\{2\sqrt{1-\cos\;\beta (\phi )}-\frac{\eta^{2}-1}{2\eta }\int_{\cos\;\beta (\phi )}^{1}\frac{dx}{\sqrt{1-x}}\left[\frac{1}{x-1∕\eta }-\frac{1}{x+1∕\eta }\right]\right\}. \end{array}$$

Note that *η* cos *β* remains greater than one for small *β* and *δ*/*R*. This point can be seen as follows. Testing if *η*^2^ cos^2^ *β* > 1 leads to testing the positivity of

$$\begin{array}{cc} \left(C^{2}+D^{2}\right) & \;\cos^{2}\beta -C^{2}=D^{2}\;\cos^{2}\beta -C^{2}\;\sin^{2}\;\beta \\ & =\sin^{2}\;\beta \left[{\left(1-\frac{\delta }{R}\right)}^{2}\cos^{2}\beta -1+2(1-\frac{\delta }{R})\;\cos\;\beta -{\left(1-\frac{\delta }{R}\right)}^{2}\;\cos^{2}\;\beta \right]\\ & =\sin^{2}\beta \left[2(1-\frac{\delta }{R})\;\cos\;\beta -1\right]>0\;\text{provided}\;(1-\frac{\delta }{R})\;\cos\;\beta >\frac{1}{2} \end{array}$$

which is easily satisfied for small *β* and *δ*/*R*. One then continues with the remaining integral in (22) by the substitution $u\equiv \sqrt{1-x}(\text{or}\;x=1-u^{2})$,

−η2−142η3C∫cosβ(ϕ)1dx1−x[1x−1∕η−1x+1∕η]=−η2−142η3C∫2sinβ(ϕ)20−2uduu[1(1−1∕η)−u2−1(1+1∕η)−u2]=−η2−142η3C[ηη−1ln1−1η+2sinβ21−1η−2sinβ2]−[ηη+1ln1+1η+2sinβ21+1n−2sinβ2]

Therefore, the first part of integral (22) (the total contribution from integral (23)) can be written as

(24) $$\begin{array}{cc} \frac{1}{2\sqrt{2}C} & \int_{\cos\;\beta (\phi )}^{1}\frac{dx}{\sqrt{1-x}}\frac{x^{2}-1}{\eta^{2}x^{2}-1}=\frac{\sin\;\frac{\beta (\phi )}{2}}{\eta^{2}C}\\ - & \frac{\eta^{2}-1}{2^{5∕2}\eta^{3}C}\left[\sqrt{\frac{\eta }{\eta -1}}\text{ln}\frac{\sqrt{1-\frac{1}{\eta }}+\sqrt{2}\;\sin\;\frac{\beta }{2}}{\sqrt{1-\frac{1}{n}}-\sqrt{2}\;\sin\;\frac{\beta }{2}}-\sqrt{\frac{\eta }{\eta +1}}\text{ln}\frac{\sqrt{1+\frac{1}{\eta }}+\sqrt{2}\;\sin\;\frac{\beta }{2}}{\sqrt{1+\frac{1}{\eta }}-\sqrt{2}\;\sin\;\frac{\beta }{2}}\right]. \end{array}$$

The second part of the integral (22) can be done in a similar fashion

(25) −122∫cosβ(ϕ)1dx1−xDx1−x2C2−(C2+D2)x2=D22C2∫cosβ(ϕ)1dxx1+xη2x2−1=D42η2C2∫cosβ(ϕ)1dx1+x[1x−1η+1x+1η]withu≡1+x=D22η2C2∫1+cosβ(ϕ)2du[u2u2−(1+1η)+u2u2−(1−1η)]=Dη2C2(1−cosβ2)+D∕C242η2[1+1ηln(2−1+1η)(2cosβ2+1+1η)(2+1+1η)(2cosβ2−1+1η)]+[1−1ηln(2−1−1η)(2cosβ2+1−1η)(2+1−1η)(2cosβ2−1−1η)].

Combining integrals (24) and (25), we have the full results for the *θ* integral (22)

(26) ∫0β(ϕ)sinθdθ22t(θ)1−cosθ=1η2C[sinβ(ϕ)2+DC(1−cosβ(ϕ)2)]+142η2C{η2−1η(η+1)ln1+1η+2sinβ21+1η−2sinβ2−η2−1η(η−1)ln1−1η+2sinβ21−1η−2sinβ2}+DC[1+1ηln(2−1+1η)(2cosβ2+1+1η)(2+1+1η)(2cosβ2−1+1η)]+{[1−1ηln(2−1−1η)(2cosβ2+1−1η)(2+1−1η)(2cosβ2−1−1η)]}.

By multiplying the result above by [(*σ*_2_ − *σ*_1_) + *λ*(*ϕ*)(*σ*_3_ − *σ*_2_)] and integrating over the *ϕ* angle from 0 to *α*, we obtain the second half of the integral (18). Prior to doing so, let us further analyze *δ*(*ϕ*) and *η* to obtain the expansion scheme. We already know that for a small spherical triangle (hence small *β*) *δ*/*R* is of order *β*^2^, *η* is of order 1/*β*, *C* is of order *β*^2^ and *D* is of order *β*. Therefore, *η*^2^*C* is of order 1. We expand expression (26) as follows.

The first and the second terms inside the curly brackets can be expanded as

(27) $$\begin{array}{cc} & 2\frac{\eta^{2}-1}{\sqrt{\eta (\eta +1)}}\left[\frac{2^{1∕2}\;\sin\;\frac{\beta }{2}}{{\left(1+\frac{1}{\eta }\right)}^{1∕2}}+\frac{2^{3∕2}\;\sin^{3∕2}\frac{\beta }{2}}{3{\left(1+\frac{1}{\eta }\right)}^{3∕2}}+\ldots \right]-2\frac{\eta^{2}-1}{\sqrt{\eta (\eta -1)}}\left[\frac{2^{1∕2}\;\sin\;\frac{\beta }{2}}{{\left(1-\frac{1}{\eta }\right)}^{1∕2}}+\frac{2^{3∕2}\;\sin^{3∕2}\frac{\beta }{2}}{{3(1-\frac{1}{\eta })}^{3∕2}}+\ldots \right]\\ & \;\;=-4\sqrt{2}\;\sin\frac{\beta }{2}\left[1+\frac{4}{3}\frac{\eta^{2}}{\eta^{2}-1}\;\sin^{2}\frac{\beta }{2}+\frac{4}{5}\frac{\eta^{2}(3\eta^{2}+1)}{{\left(\eta^{2}-1\right)}^{2}}\;\sin^{4}\frac{\beta }{2}+\ldots \right]\\ & \;\;=-4\sqrt{2}\;\sin\;\frac{\beta }{2}\left[1+\frac{4}{3}\left(1+\frac{1}{\eta^{2}}\right)\;\sin^{2}\frac{\beta }{2}+\frac{12}{5}\;\sin^{4}\frac{\beta }{2}+O(\beta^{6})\right]. \end{array}$$

Similarly, the terms inside the square brackets can be expanded as follows:

(28) $$\begin{array}{cc} \frac{D}{C} & \left[\sqrt{1+\frac{1}{\eta }}\text{ln}\frac{1-\frac{\sqrt{2}(1-\cos\;\frac{\beta }{2})}{\sqrt{2}+\sqrt{1+\frac{1}{\eta }}}}{1-\frac{\sqrt{2}(1-\cos\;\frac{\beta }{2})}{\sqrt{2}-\sqrt{1+\frac{1}{\eta }}}}+\sqrt{1-\frac{1}{\eta }}\text{ln}\frac{1-\frac{\sqrt{2}(1-\cos\;\frac{\beta }{2})}{\sqrt{2}+\sqrt{1-\frac{1}{\eta }}}}{1-\frac{\sqrt{2}(1-\cos\;\frac{\beta }{2})}{\sqrt{2}-\sqrt{1-\frac{1}{\eta }}}}\right]\\ & \;\approx \frac{D}{C}\left[2\sqrt{2}(1-\cos\frac{\beta }{2})\left(\frac{1+\frac{1}{\eta }}{1-\frac{1}{\eta }}+\frac{1-\frac{1}{\eta }}{1+\frac{1}{\eta }}\right)+4\sqrt{2}{\left(1-\cos\frac{\beta }{2}\right)}^{2}\left(\frac{1+\frac{1}{\eta }}{{\left(1-\frac{1}{\eta }\right)}^{2}}+\frac{1-\frac{1}{\eta }}{{\left(1+\frac{1}{\eta }\right)}^{2}}\right)+\ldots \right]\\ & \;\approx 4\sqrt{2}\frac{D}{C}\left[(1-\cos\frac{\beta }{2})\frac{\eta^{2}+1}{\eta^{2}-1}+2{\left(1-\cos\frac{\beta }{2}\right)}^{2}\frac{\eta^{2}(\eta^{2}+3)}{{\left(\eta^{2}-1\right)}^{2}}+{\left(1-\cos\frac{\beta }{2}\right)}^{3}\frac{2\eta^{2}}{3}\frac{7\eta^{4}+46\eta^{2}+11}{{\left(\eta^{2}-1\right)}^{3}}+\ldots \right]\\ & \;\approx 4\sqrt{2}\frac{D}{C}(1-\cos\frac{\beta }{2})\left[1+\frac{2}{\eta^{2}}+\frac{2}{\eta^{4}}+2(1-\cos\frac{\beta }{2})\left(1+\frac{5}{\eta^{2}}\right)+\frac{14}{3}{\left(1-\cos\frac{\beta }{2}\right)}^{2}+O(\beta^{6})\right]. \end{array}$$

Combining results from (26)–(28), we may write

(29) ∫0β(ϕ)sinθdθ22t(θ)1−cosθ=2η2C{DC(1−cosβ(ϕ)2)−23sin3β(ϕ)2}+DC(1−cosβ(ϕ)2)[1η2+(1−cosβ(ϕ)2)]−23η2sin3β(ϕ)2−65sin5β(ϕ)2+{DC(1−cosβ(ϕ)2)[1η4+5η2(1−cosβ(ϕ)2)+73(1−cosβ2)2]+O(β7)}.

In order to perform the *ϕ* integral, we need to express $\sin\;\frac{\beta }{2}$ and $\cos\;\frac{\beta }{2}$ in terms of *ϕ*. Using cot *β* = *a* cos(*ϕ* − *ϕ*_0_), we have

$$\frac{1}{\sin^{2}\beta }=\frac{1}{4\;\sin^{2}\frac{\beta }{2}(1-\sin^{2}\frac{\beta }{2})}=1+a^{2}\;\cos^{2}(\phi -\phi_{0}),$$

leading to

$$\begin{array}{cc} \sin\frac{\beta (\phi )}{2} & \approx \frac{1}{\sqrt{2}}{\left[1-{\left(1+\frac{1}{a^{2}\;\cos^{2}(\phi -\phi_{0})}\right)}^{-1∕2}\right]}^{1∕2}\\ & \approx \frac{1}{2a\;\cos(\phi -\phi_{0})}\left[1-\frac{3}{8}\frac{1}{a^{2}\;\cos^{2}(\phi -\phi_{0})}+\frac{31}{128}\frac{1}{a^{4}\;\cos^{4}(\phi -\phi_{0})}+\ldots \right]. \end{array}$$

This confirms that 1/(*a* cos(*ϕ* − *ϕ*_0_)) is indeed of order *β*. Similarly,

$$\cos\frac{\beta (\phi )}{2}\approx 1-\frac{1}{8a^{2}\;\cos^{2}(\phi -\phi_{0})}+\frac{11}{128a^{4}\;\cos^{4}(\phi -\phi_{0})}-\frac{69}{1024a^{6}\;\cos^{6}(\phi -\phi_{0})}+\ldots$$

or

$$1-\cos\frac{\beta (\phi )}{2}\approx \frac{1∕8a^{2}}{\cos^{2}(\phi -\phi_{0})}\left[1-\frac{11}{16a^{2}\;\cos^{2}(\phi -\phi_{0})}+\frac{69}{128a^{4}\;\cos^{4}(\phi -\phi_{0})}+\ldots \right].$$

There are a few more details we need to attend to. If we examine equation (29), we notice that there is an order 1 combination *η*^2^*C* that needs to be worked out and expanded. Also, terms inside the square brackets of equation (29), although combined to form a small contribution headed by $\left(1-\cos\;\frac{\beta }{2}\right)$, are multiplied by the overall factor *D*/*C* which is of order 1/*β* and thus we also need to work out the combination $(1-\cos\;\frac{\beta }{2})D∕C$ and expand it properly. Since the order *β*^2^ small quantity *δ*/*R* appears in *C*(*ϕ*) and *D*(*ϕ*), we also need to express adequately the leading behavior of *δ*/*R* to facilitate the proper expansions needed. We shall name the order *β*^2^ small quantity $1-{\hat{r}}_{2}\cdot {\hat{r}}_{3}$ as *ζ*_23_. To simplify our expansion, without loss of generality, we shall introduce an order 1 *ϕ*-dependent quantity

(30) $$\tilde{h}\equiv \zeta_{23}\lambda (\phi )(1-\lambda (\phi ))a^{2}\cos^{2}(\phi -\phi_{0}).$$

Specifically,

$$\begin{array}{cc} \frac{\delta (\phi )}{R} & =1-\sqrt{1-2\lambda (1-\lambda )\zeta_{23}}=\lambda (1-\lambda )\zeta_{23}\left[1+\frac{\lambda (1-\lambda )\zeta_{23}}{2}+\frac{\lambda^{2}{\left(1-\lambda \right)}^{2}\zeta_{23}^{2}}{2}+\ldots \right]\\ & \equiv \frac{\tilde{h}}{a^{2}\;\cos^{2}(\phi -\phi_{0})}[1+\frac{\tilde{h}}{2a^{2}\;\cos^{2}(\phi -\phi_{0})}+\frac{{\tilde{h}}^{2}}{2a^{4}\;\cos^{4}(\phi -\phi_{0})}+\ldots ] \end{array}$$

Similarly

$$\frac{\delta^{2}(\phi )}{R^{2}}=\frac{{\tilde{h}}^{2}}{a^{4}\;\cos^{4}(\phi -\phi_{0})}\left[1+\frac{\tilde{h}}{a^{2}\;\cos^{2}(\phi -\phi_{0})}+\ldots \right].$$

We now further examine the coefficients *C*(*ϕ*) and *D*(*ϕ*).

(31) $$\begin{array}{cc} C(\phi ) & =(1-\cos\;\beta )+\frac{\delta }{R}\cos\;\beta =(1-\cos\;\beta )+\frac{\delta }{R}-\frac{\delta }{R}(1-\cos\;\beta )\\ & =\frac{\tilde{h}+\frac{1}{2}}{a^{2}\;\cos^{2}(\phi -\phi_{0})}\left[1+\frac{2\tilde{h}-3}{4a^{2}\;\cos^{2}(\phi -\phi_{0})}+\frac{4{\tilde{h}}^{2}-4\tilde{h}+5}{8a^{4}\;\cos^{4}(\phi -\phi_{0})}+\ldots \right] \end{array}$$

and

(32) $$\begin{array}{cc} D(\phi ) & =(1-\frac{\delta }{R})\;\sin\;\beta \\ & =\frac{1}{a\;\cos(\phi -\phi_{0})}\left[1-\frac{\tilde{h}+\frac{1}{2}}{a^{2}\;\cos^{2}(\phi -\phi_{0})}-\frac{{\tilde{h}}^{2}-\tilde{h}-\frac{3}{4}}{2a^{4}\;\cos^{4}(\phi -\phi_{0})}+\ldots \right] \end{array}$$

leading to

$$\frac{D\phi }{C(\phi )}\approx \frac{2a\;\cos(\phi -\phi_{0})}{1+2\tilde{h}}\left[1+\frac{1-6\tilde{h}}{4a^{2}\;\cos^{2}(\phi -\phi_{0})}-\frac{{\left(1+2\tilde{h}\right)}^{2}}{16a^{4}\;\cos^{4}(\phi -\phi_{0})}+\ldots \right].$$

We thus have

$$\frac{D\phi }{C(\phi )}\left(1-\cos\frac{\beta }{2}\right)\approx \frac{1∕(1+2\tilde{h})}{4a\;\cos(\phi -\phi_{0})}\left\{1-\frac{24\tilde{h}+7}{16a^{2}\;\cos^{2}(\phi -\phi_{0})}-\frac{32{\tilde{h}}^{2}-100\tilde{h}-39}{128a^{4}\;\cos^{4}(\phi -\phi_{0})}+\ldots \right\}.$$

We also need to expand 1/*η*^2^ to first two leading orders. Let us begin with

(33) $$\begin{array}{cc} \eta^{2} & =\frac{C^{2}+D^{2}}{C^{2}}=1+\frac{D^{2}}{C^{2}}\\ & =\frac{4a^{2}\cos^{2}(\phi -\phi_{0})}{{\left(1+2\tilde{h}\right)}^{2}}\left[1+\frac{4{\tilde{h}}^{2}-8\tilde{h}+3}{4a^{2}\cos^{2}(\phi -\phi_{0})}+\frac{28{\tilde{h}}^{2}-20\tilde{h}-1}{16a^{4}\cos^{4}(\phi -\phi_{0})}+\ldots \right]. \end{array}$$

Therefore, we have

$$\frac{1}{\eta^{2}}=\frac{{\left(\tilde{h}+\frac{1}{2}\right)}^{2}}{a^{2}\cos^{2}(\phi -\phi_{0})}\left[1-\frac{4{\tilde{h}}^{2}-8\tilde{h}+3}{4a^{2}\cos^{2}(\phi -\phi_{0})}+\frac{8{\tilde{h}}^{4}-32{\tilde{h}}^{3}+30{\tilde{h}}^{2}-14\tilde{h}+5}{8a^{4}\cos^{4}(\phi -\phi_{0})}+\ldots \right].$$

Also, the order 1 quantity 2/*η*^2^*C* may also be expanded as

$$\frac{2}{\eta^{2}C}=(2\tilde{h}+1)\left\{1-\frac{\tilde{h}(\tilde{h}-\frac{3}{2})}{a^{2}\cos^{2}(\phi -\phi_{0})}+\frac{\tilde{h}\left(8{\tilde{h}}^{3}-28{\tilde{h}}^{2}+14\tilde{h}-1\right)}{8a^{4}\cos^{4}(\phi -\phi_{0})}+\ldots \right\},$$

and finally the expression inside the curly brackets of equation (29), after some tedious algebra, may be expanded in powers of 1/*a* cos(*ϕ* − *ϕ*_0_) as

$$\frac{1∕(1+2\tilde{h})}{a\;\cos(\phi -\phi_{0})}\left[\frac{1}{4}+\frac{48{\tilde{h}}^{2}-56\tilde{h}-19}{192\;a^{2}\cos^{2}(\phi -\phi_{0})}+\frac{1600{\tilde{h}}^{3}-120{\tilde{h}}^{2}+1164\tilde{h}+507}{7680\;a^{4}\cos^{4}(\phi -\phi_{0})}+\ldots \right].$$

We may now rewrite the expression (29) as

(34) ∫0β(ϕ)sinθdθ22t(θ)1−cosθ=(2h~+1)[1−h~(h~−32)a2cos2(ϕ−ϕ0)+h~(8h~3−28h~2+14h~−1)8a4cos4(ϕ−ϕ0)+…]×1∕(1+2h~)acos(ϕ−ϕ0)[14+48h~2−56h~−19192a2cos2(ϕ−ϕ0)+1600h~3−120h~2+1164h~+5077680a4cos4(ϕ−ϕ0)+…]=1acos(ϕ−ϕ0){14+16h~−19192a2cos2(ϕ−ϕ0)+640h~2−216h~+5077680a4cos4(ϕ−ϕ0)}+{186368h~3−47488h~2+29280h~−876371720320a6cos6(ϕ−ϕ0)+O(β8)}.

Note that the order 1 variable $\tilde{h}$ depends on the azimuthal angle *ϕ*. The expression above will be multiplied by [(*σ*_2_ − *σ*_1_) + *λ*(*ϕ*)(*σ*_3_ − *σ*_2_)] and then integrated over *ϕ* from 0 to *α*. In equation (34), we have kept terms all the way up to *β*^7^. However, as a proof of principle we will only keep terms up to *β*^3^ in the remainder of the manuscript. (Although the systematic approach to reach higher order terms will be described, we note that the *ϕ* integrals can be quite tedious for higher order terms.) Given $\tilde{h}\equiv \zeta_{23}\lambda (\phi )(1-\lambda (\phi ))a^{2}\;\cos^{2}(\phi -\phi_{0})$, integrals of the following sorts (*ϕ* dependence of *λ* suppressed) must appear

(35) $$F(\ell ,d,p)\equiv \int_{0}^{\alpha }{\tilde{h}}^{\ell }\lambda^{d}\frac{1}{a^{p}\cos^{p}(\phi -\phi_{0})}d\phi =\zeta_{23}^{\ell }a^{2\ell -p}\int_{0}^{\alpha }\lambda^{\ell +d}{\left(1-\lambda \right)}^{\ell }\cos^{2\ell -p}(\phi -\phi_{0})d\phi$$

with *d* = 0 or 1, *p* an odd positive integer, and the nonnegative integer *ℓ* < *p*/2. The case of *ℓ* = *d* = 0 has been worked out explicitly in (21). Therefore, we concentrate here on *ℓ* + *d* ⩾ 1, meaning the integral exponent of *λ* is positive.

From equation (12), we see that both *λ* and 1 − *λ* are rational function of sin *ϕ* and cos *ϕ*. If one substitutes $\tan\;\frac{\phi }{2}$ by *x*, then *λ* becomes a rational function of *x* and $d\phi =\frac{2\;dx}{1+x^{2}}$. We write

$$\begin{array}{cc} \lambda & =\frac{\sin\;\beta_{3}\;\sin\;\phi }{\sin\;\beta_{2}\;\sin(\alpha -\phi )+\sin\;\beta_{3}\sin\;\phi }\equiv \frac{A\;\sin\;\phi }{B_{1}\;\cos\;\phi +(A+B_{2})\;\sin\;\phi }\\ 1-\lambda & =\frac{\sin\;\beta_{2}\;\sin(\alpha -\phi )}{\sin\;\beta_{2}\;\sin(\alpha -\phi )+\sin\;\beta_{3}\;\sin\;\phi }\equiv \frac{B_{1}\;\cos\;\phi +B_{2}\;\sin\;\phi }{B_{1}\;\cos\;\phi +(A+B_{2})\;\sin\;\phi } \end{array}$$

with *A* ≡ sin *β*_3_, *B*_1_ = sin *β*_2_ sin *α*, and *B*_2_ = −sin *β*_2_ cos *α*. Upon setting $\tan\;\frac{\phi }{2}=x$, we have

$$\sin\;\phi =\frac{2x}{1+x^{2}}\;\;\;\text{and}\;\;\;\cos\;\phi =\frac{1-x^{2}}{1+x^{2}},$$

and consequently,

$$\begin{array}{cc} \lambda = & \frac{2\mathrm{Ax}}{B_{1}(1-x^{2})+2x(A+B_{2})}=\frac{2\;\sin\;\beta_{3}}{\sin\;\beta_{2}\;\sin\;\alpha }\frac{x}{1-x^{2}+2\frac{A+B_{2}}{B_{1}}x}\\ 1-\lambda = & \frac{B_{1}(1-x^{2})+2B_{2}x}{B_{1}(1-x^{2})+2x(A+B_{2})}=\frac{1-x^{2}-2x\;\cot\;\alpha }{1-x^{2}+2\frac{A+B_{2}}{B_{1}}x}. \end{array}$$

We also write

$$\cos(\phi -\phi_{0})=\cos\;\phi_{0}\;\cos\;\phi +\sin\;\phi_{0}\;\sin\;\phi =\frac{\cos\;\phi_{0}}{1+x^{2}}[1-x^{2}+2x\;\tan\;\phi_{0}].$$

It turns out that tan *ϕ*_0_ and (*A* + *B*_2_)/*B*_1_ are very close when *β* ≪ 1. From equation (4)

$$\tan\;\phi_{0}=\frac{\frac{\tan\;\beta_{3}}{\tan\;\beta_{2}}-\cos\;\alpha }{\sin\;\alpha },$$

while

$$\frac{A+B_{2}}{B_{1}}=\frac{\sin\;\beta_{3}-\sin\;\beta_{2}\;\cos\;\alpha }{\sin\;\beta_{2}\;\sin\;\alpha }=\frac{\frac{\sin\;\beta_{3}}{\sin\;\beta_{2}}-\cos\;\alpha }{\sin\;\alpha }.$$

Evidently, $\frac{A+B_{2}}{B_{1}}-\tan\;\phi_{0}=\frac{\sin\;\beta_{3}}{\sin\;\beta_{2}\;\sin\;\alpha }(1-\frac{\cos\;\beta_{2}}{\cos\;\beta_{3}})$ is of order *β*^2^ when *β*_2_ and *β*_3_ are small. To simplify the operation, we introduce the following shorthand notations

$$\begin{array}{cc} \mu = & \frac{B_{2}}{B_{1}}=-\cot\;\alpha \\ \tau = & \tan\;\phi_{0}=\frac{\frac{\tan\;\beta_{3}}{\tan\;\beta_{2}}}{\sin\;\alpha }+\mu \\ \tau^{\prime }= & \frac{A+B_{2}}{B_{1}}=\frac{\frac{\sin\;\beta_{3}}{\sin\;\beta_{2}}}{\sin\;\alpha }+\mu \end{array}$$

and

$$\tau^{1}-\tau =\frac{\sin\;\beta_{3}}{\sin\;\beta_{2}\;\sin\;\alpha }(1-\frac{\cos\;\beta_{2}}{\cos\;\beta_{3}})\propto O(\beta^{2}).$$

Below we will illustrate the basic idea how the order of *β* are incorporated in F(ℓ,d,p) when *ℓ* + *d* ⩾ 1. We write (with $x\equiv \tan\;\frac{\phi }{2}$)

$$\begin{array}{cc} F(\ell ,d,p)= & \zeta_{23}^{\ell }a^{2\ell -p}\int_{0}^{\alpha }\lambda^{\ell +d}{\left(1-\lambda \right)}^{\ell }\cos^{2\ell -p}(\phi -\phi_{0})d\phi \\ & =2\zeta_{23}^{\ell }{\left(\frac{2\;\sin\;\beta_{3}}{\sin\;\beta_{2}\;\sin\;\alpha }\right)}^{\ell +d}{\left(a\;\cos\;\phi_{0}\right)}^{2\ell -p}\int_{0}^{\tan\;\frac{\alpha }{2}}dx\frac{x^{\ell +d}{\left[1-x^{2}+2\mu x\right]}^{\ell }{\left[1-x^{2}+2\tau x\right]}^{2\ell -p}}{{\left[1-x^{2}+2\tau^{\prime }x\right]}^{2\ell +d}{\left(1+x^{2}\right)}^{2\ell -p+1}}. \end{array}$$

By writing *τ*′ = *τ* + (*τ*′ − *τ*), we may expand part of the integrand of the above integral as

$$\frac{1}{{\left[1-x^{2}+2\tau^{\prime }x\right]}^{\left(2\ell +d\right)}}=\frac{1}{{\left[1-x^{2}+2\tau x\right]}^{\left(2\ell +d\right)}}\left(\sum_{k=0}^{\infty }C_{k}^{-(2\ell +d)}{\left[\frac{2(\tau^{\prime }-\tau )x}{1-x^{2}+2\tau x}\right]}^{k}\right)$$

where the negative binomial coefficient $C_{k}^{-n}$ (with *n* ⩾ 1) is a short hand for

$$C_{k}^{-n}\equiv \frac{\left(-n)(-n-1)\ldots (-n-k+1\right)}{k!}={\left(-1\right)}^{k}\frac{(k+n-1)!}{k!(n-1)!}={\left(-1\right)}^{k}C_{k}^{k+n-1}.$$

Consequently,

$$\begin{array}{cc} F(\ell ,d,p)= & 2\zeta_{23}^{\ell }{\left(\frac{2\;\sin\;\beta_{3}}{\sin\;\beta_{2}\;\sin\;\alpha }\right)}^{\ell +d}{\left(a\;\cos\;\phi_{0}\right)}^{2\ell -p}\\ & \times \left\{\sum_{k=0}^{\infty }{\left(-1\right)}^{k}C_{k}^{2\ell +d+k-1}2^{k}{\left(\tau^{\prime }-\tau \right)}^{k}\int_{0}^{\tan\;\frac{\alpha }{2}}\frac{x^{\ell +d+k}{\left[1-x^{2}+2\mu x\right]}^{\ell }dx}{{\left[1-x^{2}+2\tau x\right]}^{p+d+k}{\left(1+x^{2}\right)}^{2\ell -p+1}}\right\}. \end{array}$$

With (*τ*′ − *τ*) being of order $O(\beta^{2})$, the above expansion of F provides a systematic inclusion of higher order *β* contributions. We should note that the factor 1 − *x*^2^ + 2*τx* is of order 1 and is always large compared to *β* in the range 0 ⩽ *ϕ* ⩽ *α*. Given that *f*(*x*) ≡ 1 − *x*^2^ + 2*τx* is a downward parabola in *x*, we know that $f(x)\geq \min\{f(0),f(\tan\;\frac{\alpha }{2})\}$. Since *f*(0) = 1 and $f(\tan\;\frac{\alpha }{2})=\frac{\tan\;\beta_{3}}{\tan\;\beta_{2}}∕\cos^{2}\frac{\alpha }{2}$, both order 1 quantities, we find (1 − *x*^2^ + 2*τx*) always much larger than *β*. We find it more convenient to define the following integral

(36) $$\begin{array}{cc} G(\ell ,d,k,p)\equiv & 2\zeta_{23}^{\ell }{\left(\frac{2\;\sin\;\beta_{3}}{\sin\;\beta_{2}\;\sin\;\alpha }\right)}^{\ell +d+k}{\left(a\;\cos\;\phi_{0}\right)}^{2\ell -p}{\left(-1\right)}^{k}C_{k}^{2\ell +d+k-1}{\left(1-\frac{\cos\;\beta_{2}}{\cos\;\beta_{3}}\right)}^{k}\\ & \times \int_{0}^{\tan\;\frac{\alpha }{2}}\frac{x^{\ell +d+k}{\left[1-x^{2}+2\mu x\right]}^{\ell }dx}{{\left[1-x^{2}+2\tau x\right]}^{p+d+k}{\left(1+x^{2}\right)}^{2\ell -p+1}}. \end{array}$$

(Evidently, because of the prefactors (*a*^2^*ζ*_23_)^*ℓ*^*a*^−p^ and ${\left(1-\frac{\cos\;\beta_{2}}{\cos\;\beta_{3}}\right)}^{k}$, G(ℓ,d,k,p) is $O(\beta^{p+2k})$.) Then for *ℓ* + *d* ⩾ 1, we can write

(37) $$F(\ell ,d,p)=\sum_{k=0}^{\infty }G(\ell ,d,k,p).$$

For future convenience, let us generalize the integral above to

(38) $$F_{n_{c},n_{s}}(\ell ,d,p)\equiv \int_{0}^{\alpha }{\tilde{h}}^{\ell }\lambda^{d}\frac{\cos^{n_{c}}\phi \;\sin^{n_{s}}\;\phi }{a^{p}\;\cos^{p}(\phi -\phi_{0})}d\phi ,$$

leading naturally to

(39) $$\begin{array}{cc} G_{n_{c},n_{s}}(\ell ,d,k,p)\equiv & 2\zeta_{23}^{\ell }{\left(\frac{2\;\sin\;\beta_{3}}{\sin\;\beta_{2}\;\sin\;\alpha }\right)}^{\ell +d+k}{\left(a\;\cos\;\phi_{0}\right)}^{2\ell -p}{\left(-1\right)}^{k}C_{k}^{2\ell +d+k-1}{\left(1-\frac{\cos\;\beta_{2}}{\cos\;\beta_{3}}\right)}^{k}\\ & \times \int_{0}^{\tan\;\frac{\alpha }{2}}\frac{2^{n_{s}}x^{\ell +d+k+n_{s}}{\left(1-x^{2}\right)}^{n_{c}}{\left[1-x^{2}+2\mu x\right]}^{\ell }dx}{{\left[1-x^{2}+2\tau x\right]}^{p+d+k}{\left(1+x^{2}\right)}^{2\ell -p+1+n_{c}+n_{s}}} \end{array}$$

and

(40) $$F_{n_{c},n_{s}}(\ell ,d,p)=\sum_{k=0}^{\infty }G_{n_{c},n_{s}}(\ell ,d,k,p).$$

The leading term of $F_{n_{c},n_{s}}(\ell ,d,p)$, $G_{n_{c},n_{s}}(\ell ,d,k=0,p)$ is $O(\beta^{p})$, and each subsequent *k* ≠ 0 term carries an additional *β*^2*k*^ order. As an example of the use of this information, to retain order $O(\beta^{5})$ accuracy in F(ℓ,d,p)s, we need only the leading (*k* = 0) terms for F(ℓ,d,p=5)s, both the leading and the subleading (*k* = 1) terms for F(ℓ,d,p=3)s, and the first three leading terms (*k* = 0, 1, 2) for F(ℓ,d,p=1)s. Specifically, we employ the following approximations

$$\begin{array}{cc} F_{n_{c},n_{s}}(\ell ,d,p=5) & \approx G_{n_{c},n_{s}}(\ell ,d,0,5)\\ F_{n_{c},n_{s}}(\ell ,d,p=3) & \approx G_{n_{c},n_{s}}(\ell ,d,0,3)+G_{n_{c},n_{s}}(\ell ,d,1,3)\\ F_{n_{c},n_{s}}(\ell ,d,p=1) & \approx G_{n_{c},n_{s}}(\ell ,d,0,1)+G_{n_{c},n_{s}}(\ell ,d,1,1)+G_{n_{c},n_{s}}(\ell ,d,2,1). \end{array}$$

However, we also note that

(41) $$F_{n_{c},n_{s}}(\ell =0,d=0,p)=G_{n_{c},n_{s}}(0,0,0,p)$$

with no higher order correction.

Because the integrands are all rational functions of *x*, they can be integrated exactly using, for example, the Ostrogradskiy–Hermite method. In appendix C, we list some results of these integrals.

Note that *σ*_*i*_ − *σ*_*j*_ (when *i* and *j* are neighboring vertices and when the spherical triangles are small) should be of order *β* based on the charge distribution continuity. (We may imagine Taylor expanding the charge distribution around a fixed point (say vertex *i* of the triangle considered.) Using equations (19) and (34), the electric field contribution in equation (18) accurate up to order $O(\beta^{5})$ can be written as

Ez=∫0αdϕ∫0β(ϕ)sinθdθ22σ1+t(θ)[σ2−σ1+λ(ϕ)(σ3−σ2)]1−cosθ=σ1[F(0,0,1)2−F(0,0,3)16∕3+F(0,0,5)256∕31]+(σ2−σ1)[F(0,0,1)4−F(0,0,3)192∕19+F(1,0,3)12]+(σ3−σ2)[F(0,1,1)4−F(0,1,3)192∕19+F(1,1,3)12]=σ1[G(0,0,0,1)2−G(0,0,0,3)16∕3+G(0,0,0,5)256∕31]+(σ2−σ1)[G(0,0,0,1)4−G(0,0,0,3)192∕19+G(1,0,0,3)12]+(σ3−σ2)[G(0,1,0,1)+G(0,1,1,1)4]−[G(0,1,0,3)192∕19+G(1,1,0,3)12].

Looking up the integral results from appendix C, one sees that the expression becomes complicated as one goes to higher order terms. However, we have made an effort to keep most of the results expanded in powers of cos *ϕ*_0_ and cos(*α* *λ* *ϕ*_0_) although the expressions may still contain a single power of sin *ϕ*_0_ and/or sin(*α* − *ϕ*_0_). This choice is for computational convenience as one only need to obtain cos *ϕ*_0_ (or cos(*α* − *ϕ*_0_)) once and then store it and raise it to any power as needed. Even though one may in principle keep as many higher order terms as one wishes, for illustration purpose, we will from this point on only keep to order *β*_3_ accuracy as previously mentioned. By looking at the development up to this point, the readers would have gotten the idea of how to keep higher order terms when needed. Remember that G(ℓ,d,k,p) is of order *β*^*p*+2*k*^ and that (*σ*_2_ − *σ*_1_)/*σ*^1(2)^ is of order *β*, we therefore have (with accuracy up to order *β*^3^)

(42) $$\begin{array}{cc} E_{z} & =\int_{0}^{\alpha }d\phi \int_{0}^{\beta (\phi )}\frac{\sin\;\theta \;d\theta }{2\sqrt{2}}\frac{\sigma_{1}+t(\theta )[\sigma_{2}-\sigma_{1}+\lambda (\phi )(\sigma_{3}-\sigma_{2})]}{\sqrt{1-\cos\;\theta }}\\ & \approx \sigma_{1}\left[\frac{G(0,0,0,1)}{2}-\frac{G(0,0,0,3)}{16∕3}\right]+(\sigma_{2}-\sigma_{1})\frac{G(0,0,0,1)}{4}+(\sigma_{3}-\sigma_{2})\frac{G(0,1,0,1)}{4}+O(\beta^{4})\\ & =\frac{\sigma_{1}+\sigma_{2}}{4}G(0,0,0,1)-\frac{3\sigma_{1}}{16}G(0,0,0,3)+\frac{\sigma_{3}-\sigma_{2}}{4}G(0,1,0,1)+O(\beta^{4})\\ & \approx \left[\frac{\sigma_{1}+\sigma_{2}}{8a}-\frac{17\sigma_{1}+19\sigma_{2}}{192\cdot 4a^{3}}+\frac{\left(\sigma_{3}-\sigma_{2})\sin\;\beta_{3}\;\sin(2\phi_{0}\right)}{16a\sin\;\alpha \;\sin\;\beta_{2}}\right]\text{ln}\frac{\left[1+\sin(\alpha -\phi_{0})][1+\sin(\phi_{0})\right]}{\left[1-\sin(\alpha -\phi_{0})][1-\sin(\phi_{0})\right]}\\ & \;\;-\frac{17\sigma_{1}+19\sigma_{2}}{192\cdot 2a^{3}}\left(\frac{\sin(\alpha -\phi_{0})}{\cos^{2}(\alpha -\phi_{0})}+\frac{\sin\;\phi_{0}}{\cos^{2}\;\phi_{0}}\right)\\ & \;\;+\frac{(\sigma_{3}-\sigma_{2})\;\cos\;\beta_{3}}{4a\;\sin\;\alpha \;\cos\;\beta_{2}}[\cos\;\phi_{0}-\cos(\alpha -\phi_{0})]+O(\beta^{4}). \end{array}$$

We can thus compute the field contribution of all spherical triangles containing the north pole as the one of the vertices. Once contributions of all those spherical triangles are summed, we still need to remember to add 2*πσ*_1_ to the field right above the north pole and −2*πσ*_1_ to the field right below the north pole.

### 3.5. Radial electric field on the same sphere but outside the charged spherical triangle

Now consider the radial electric field, produced by the spherical triangle discussed earlier, at a point outside the solid angle region of the spherical triangle. Let us denote the radial direction by the polar angles (*θ*, *ϕ*). For *α* < *ϕ* < 2*π*, *θ* can take the full range 0 ⩽ *θ* ⩽ *π*. However, when 0 ⩽ *ϕ* ⩽ *α*, the range of *θ* becomes

$$\tan^{-1}\left(\frac{1}{a\;\cos(\phi -\phi_{0})}\right)⩽\theta ⩽\pi .$$

Remember

$$\frac{1}{|\tilde{r}-r|}=\sum_{l=0}^{\infty }\frac{r_{<}^{l}}{r_{>}^{l+1}}\frac{4\pi }{2l+1}\sum_{m=-l}^{l}Y_{lm}(\hat{\tilde{r}})Y_{lm}^{*}(\hat{r})$$

where $r_{<}=\min(\tilde{r},r)$, $\hat{r}\equiv r∕r$, and in spherical polar coordinates $\hat{r}\equiv (\theta ,\phi )$.

Electric potential produced by the spherical triangle (with 0 ⩽ *ϕ* ⩽ *α* and 0 ⩽ *θ* ⩽ tan^−1^ $\left(\frac{1}{a\;\cos(\phi -\phi_{0})}\right)$) at location $\tilde{r}$ can be written as

$$\int_{0}^{\alpha }d\phi \int_{0}^{\beta (\phi )}\sin\;\theta d\theta \frac{\sigma (\theta ,\phi )}{|\tilde{r}-r|}R^{2}=\sum_{l=0}^{\infty }\frac{r_{<}^{l}}{r_{>}^{l+1}}\frac{4\pi R^{2}}{2l+1}\sum_{m=-l}^{l}Y_{lm}(\tilde{\theta },\tilde{\phi })\int_{0}^{\alpha }d\phi \int_{0}^{\beta (\phi )}\sin\;\theta d\theta \;Y_{lm}^{*}(\theta ,\phi )\sigma (\theta ,\phi ).$$

The electric field at the spherical surface will be needed when enforcing the boundary condition. If we take the derivative by assuming first $\tilde{r}>R$, we obtain

$$E_{\hat{r}}(\tilde{r}=R)=\sum_{l=0}^{\infty }\frac{4\pi (l+1)}{2l+1}\sum_{m=-l}^{l}Y_{lm}(\tilde{\theta },\tilde{\phi })\int_{0}^{\alpha }d\phi \int_{0}^{\beta (\phi )}\sin\;\theta d\theta \;Y_{lm}^{*}(\theta ,\phi )\sigma (\theta ,\phi ).$$

If we take the derivative by assuming first $\tilde{r}<R$, we obtain

$$E_{\hat{r}}(\tilde{r}=R)=\sum_{l=0}^{\infty }\frac{4\pi l}{2l+1}\sum_{m=-l}^{l}Y_{lm}(\tilde{\theta },\tilde{\phi })\int_{0}^{\alpha }d\phi \int_{0}^{\beta (\phi )}\sin\;\theta d\theta \;Y_{lm}^{*}(\theta ,\phi )\sigma (\theta ,\phi ).$$

These two expressions suggest that

$$\begin{array}{cc} & \sum_{l=0}^{\infty }\frac{4\pi (l+1)}{2l+1}\sum_{m=-l}^{l}Y_{lm}(\tilde{\theta },\tilde{\phi })Y_{lm}^{*}(\theta ,\phi )=-\sum_{l=0}^{\infty }\frac{4\pi l}{2l+1}\sum_{m=-l}^{l}Y_{lm}((\tilde{\theta },\tilde{\phi })Y_{lm}^{*}(\theta ,\phi )\;\text{or}\\ & \sum_{l=0}^{\infty }\sum_{m=-l}^{l}Y_{lm}(\tilde{\theta },\tilde{\phi })Y_{lm}^{*}(\theta ,\phi )=\langle \hat{\tilde{r}}|\left\{\sum_{l,m}|l,m\rangle \langle l,m|\right\}|\hat{r}\rangle =\langle \hat{\tilde{r}}|\hat{r}\rangle =0. \end{array}$$

This is correct since direction of $\tilde{r}$ is outside the spherical triangle while the direction of **r** is within the spherical triangle. Therefore, $\hat{\tilde{r}}\neq \hat{r}$ always. This indicates that we can actually average the two expressions for $E_{\hat{r}}$ above to arrive at

(43) $$\begin{array}{cc} E_{\hat{r}}(r=R) & =\sum_{l=0}^{\infty }\frac{2\pi }{2l+1}\sum_{m=-l}^{l}Y_{lm}(\tilde{\theta },\tilde{\phi })\int_{0}^{\alpha }d\phi \int_{0}^{\beta (\phi )}\sin\;\theta d\theta \;Y_{lm}^{*}(\theta ,\phi )\sigma (\theta ,\phi )\\ & =\frac{1}{2}\int_{0}^{\alpha }d\phi \int_{0}^{\beta (\phi )}\sin\;\theta d\theta \frac{R^{2}\sigma (\theta ,\phi )}{R^{2}\sqrt{2-2\hat{\tilde{r}}\cdot \hat{r}}}. \end{array}$$

The correctness of the expression above can be verified by direct evaluation. Let us consider the radial field produced at location $\tilde{r}$ by a charge *q_t_* at **r**:

$$q_{t}\frac{\tilde{r}-r}{|\tilde{r}-r{|}^{3}}\cdot \hat{\tilde{r}}=q_{t}\frac{\tilde{r}-r\;\hat{\tilde{r}}\cdot \hat{r}}{({\tilde{r}}^{2}+r^{2}-2\tilde{r}r\;\hat{\tilde{r}}\cdot \hat{r}{)}^{3∕2}}.$$

Upon setting $r=\tilde{r}=R$, we obtain

$$\frac{q_{t}}{2R^{2}}\frac{1}{\sqrt{2-2\hat{\tilde{r}}\cdot \hat{r}}}$$

ni exact agreement with (43). This is a great simplification as the radial electric field expression (43) happens to be $\frac{1}{2R}$ times the electric potential.

In the previous subsection, the electric field at the north pole was computed using *β* (the range of polar *θ* angle) as the smallness parameter. In fact, the leading term in electric field is of order *β* and we worked out its leading correction explicitly to $O(\beta^{3})$, that is *β*^2^ smaller than the leading term. Our case here is slightly different. Previously, we have $\tilde{r}\|\hat{z}$ and thus $1∕\sqrt{1-\hat{\tilde{r}}\cdot \hat{r}}=1∕\sqrt{1-\cos\;\theta }\propto 1∕\sin\;\frac{\theta }{2}$, which got cancelled by the Jacobian factor $\sin\;\theta =2\;\sin\;\frac{\theta }{2}\;\cos\;\frac{\theta }{2}$ and the integral is well behaved. In the current case, the factor

$$\frac{1}{\sqrt{1-\hat{\tilde{r}}\cdot \hat{r}}}=\frac{1}{\sqrt{1-[\cos\;\tilde{\theta }\;\cos\;\theta +\sin\;\tilde{\theta }\;\sin\;\theta \;\cos(\tilde{\phi }-\phi )]}}$$

presents some difficulty for integration over *θ* and *ϕ*. Specifically, there is no clean way to perform an expansion. For example, although we know for sure $-1<\hat{\tilde{r}}\cdot \hat{r}<1$, expanding $1∕\sqrt{1-\hat{\tilde{r}}\cdot \hat{r}}$ using $\hat{\tilde{r}}\cdot \hat{r}$ as the expansion parameter can be extremely slow converging when $\tilde{\theta }$ is small because the integration domain is an area close to and include the north pole. To draw a close analog to the previous case, we write

(44) $$\begin{array}{cc} \frac{1}{\sqrt{1-\hat{\tilde{r}}\cdot \hat{r}}} & =\frac{1}{\sqrt{1-\hat{\tilde{r}}\cdot {\hat{r}}_{\text{ave}}-\hat{\tilde{r}}\cdot [\hat{r}-{\hat{r}}_{\text{ave}}]}}=\frac{1}{\sqrt{1-\hat{\tilde{r}}\cdot {\hat{r}}_{\text{ave}}}}{\left[1-\frac{\hat{\tilde{r}}\cdot [\hat{r}-{\hat{r}}_{\text{ave}}]}{1-\hat{\tilde{r}}\cdot {\hat{r}}_{\text{ave}}}\right]}^{-1∕2}\\ & =\frac{1}{\sqrt{1-\hat{\tilde{r}}\cdot {\hat{r}}_{\text{ave}}}}\left[1+\frac{\hat{\tilde{r}}\cdot [\hat{r}-{\hat{r}}_{\text{ave}}]}{2(1-\hat{\tilde{r}}\cdot {\hat{r}}_{\text{ave}})}+\frac{3}{8}{\left(\frac{\hat{\tilde{r}}\cdot [\hat{r}-{\hat{r}}_{\text{ave}}]}{1-\hat{\tilde{r}}\cdot {\hat{r}}_{\text{ave}}}\right)}^{2}+\frac{5}{16}{\left(\frac{\hat{\tilde{r}}\cdot [\hat{r}-{\hat{r}}_{\text{ave}}]}{1-\hat{\tilde{r}}\cdot {\hat{r}}_{\text{ave}}}\right)}^{3}+\cdots \right] \end{array}$$

with ${\hat{r}}_{\text{ave}}$ representing the average of $\hat{r}=(\sin\;\theta \;\cos\;\phi ,\sin\;\theta \;\sin\;\phi ,\cos\;\theta )$ over the integration domain and $\frac{\hat{\tilde{r}}\cdot [\hat{r}-{\hat{r}}_{\text{ave}}]}{1-\hat{\tilde{r}}\cdot {\hat{r}}_{\text{ave}}}$ as the expansion parameter. This choice bears resemblance to the usual multipole expansion. Evidently, we need $1-\hat{\tilde{r}}\cdot {\hat{r}}_{\text{ave}}$ to be significantly larger than $\hat{\tilde{r}}\cdot [\hat{r}-{\hat{r}}_{\text{ave}}]$ in order for the series to converge fast. A simple analysis shows that if

(45) $$1-\frac{1}{3}\hat{\tilde{r}}\cdot ({\hat{r}}_{1}+{\hat{r}}_{2}+{\hat{r}}_{1})>2[1-\text{min}\{{\hat{r}}_{1}\cdot {\hat{r}}_{2},{\hat{r}}_{1}\cdot {\hat{r}}_{3},{\hat{r}}_{2}\cdot {\hat{r}}_{3}\}]$$

then the expansion (44) converges reasonably fast. However, if the condition (45) is not met, it is best to integrate numerically for that particular $\hat{\tilde{r}}$.

To proceed with the expansion, we need to first find the average ${\hat{r}}_{\text{ave}}=(\bar{\sin\;\theta \;\cos\;\phi },\bar{\sin\;\theta \;\sin\;\phi },\bar{\cos\;\theta })\equiv (t_{x},t_{y},t_{z})$ over the integration domain $\int_{0}^{\alpha }d\phi \int_{0}^{\beta (\phi )}\sin\;\theta d\theta$. Before we perform the averages one by one, let us first find out the area of the domain of integration, which we will need when computing the average. (Let *y* = sin(*ϕ* − *ϕ*_0_) be the substitution variable below.)

(46) $$\begin{array}{cc} A & =\int_{0}^{\alpha }d\phi \int_{0}^{\beta (\phi )}\sin\;\theta d\theta =\int_{0}^{\alpha }d\phi [1-\cos\;\beta (\phi )]=\int_{0}^{\alpha }d\phi \left[1-\frac{a\;\cos(\phi -\phi_{0})}{\sqrt{1+a^{2}\;\cos^{2}(\phi -\phi_{0})}}\right]\\ & =\alpha -\int_{-\sin\;\phi_{0}}^{\sin(\alpha -\phi_{0})}\frac{a\;dy}{\sqrt{(1+a^{2})-a^{2}y^{2}}}\\ & =\alpha -\left[\tan^{-1}\frac{a\;\sin(\alpha -\phi_{0})}{\sqrt{1+a^{2}\;\cos^{2}(\alpha -\phi_{0})}}+\tan^{-1}\frac{a\;\sin\;\phi_{0}}{\sqrt{1+a^{2}\;\cos^{2}\;\phi_{0}}}\right]\\ & =\alpha -\tan^{-1}[\tan(\alpha -\phi_{0})\;\cos\;\beta_{2}]-\tan^{-1}[\tan(\phi_{0})\;\cos\;\beta_{3}]\\ & \approx (1-\cos\;\beta_{2})\;\sin(\alpha -\phi_{0})\;\cos(\alpha -\phi_{0})+(1-\cos\;\beta_{3})\;\sin\;\phi_{0}\;\cos\;\phi_{0}\\ & \;\;+(1-\cos\;\beta_{2}{)}^{2}\;\sin^{3}(\alpha -\phi_{0})\;\cos(\alpha -\phi_{0})+(1-\cos\;\beta_{3}{)}^{2}\;\sin^{3}\;\phi_{0}\;\cos\;\phi_{0}+O(\beta^{6}). \end{array}$$

We now calculate the average below

$$\begin{array}{cc} \bar{\sin\;\theta \;\cos\;\phi }\;A & =\int_{0}^{\alpha }\cos\;\phi \;d\phi \int_{0}^{\beta (\phi )}\sin^{2}\;\theta \;d\theta =\frac{1}{2}\int_{0}^{\alpha }\cos\;\phi \;[\beta (\phi )-\sin\;\beta (\phi )\;\cos\;\beta (\phi )]d\phi \\ & =\frac{1}{2}\int_{0}^{\alpha }\cos\;\phi \left[\tan^{-1}\frac{1}{a\;\cos(\phi -\phi_{0})}-\frac{a\;\cos\;(\phi -\phi_{0})}{1+a^{2}\;\cos^{2}(\phi -\phi_{0})}\right]d\phi . \end{array}$$

We then perform integration by parts only for the first term inside the square brackets and great simplification results

(47) $$\begin{array}{cc} \bar{\sin\;\theta \;\cos\;\phi }\;A & =\frac{1}{2}\left\{{\left[\sin\;\phi \;\tan^{-1}\frac{1}{a\;\cos\;(\phi -\phi_{0})}\right]}_{0}^{\alpha }-\int_{0}^{\alpha }\frac{a\;\cos\;\phi_{0}\;d\phi }{1+a^{2}\;\cos(\phi -\phi_{0})}\right\}\\ & =\frac{1}{2}\left\{\sin\;\alpha \;\tan^{-1}\frac{1}{a\;\cos(\alpha -\phi_{0})}-\frac{a\;\cos\;\phi_{0}}{\sqrt{1+a^{2}}}\left[\tan^{-1}\frac{\tan(\alpha -\phi_{0})}{\sqrt{1+a^{2}}}+\tan^{-1}\frac{\tan\;(\phi_{0})}{\sqrt{1+a^{2}}}\right]\right\}\\ & =\frac{1}{2}\left\{\beta_{2}\;\sin\;\alpha \left[1-\frac{\sin\;\beta_{2}}{\beta_{2}}\frac{\beta_{1}}{\sin\;\beta_{1}}\;\cos\;\beta_{3}\right]\right\} \end{array}$$

where equations (8) and (9) have been applied to simplify the expression above.

Similarly,

(48) sinθsinϕ‾A=12∫0αsinϕ[tan−11acos(ϕ−ϕ0)−acos(ϕ−ϕ0)1+a2cos2(ϕ−ϕ0)]dϕ=12{cot−1(acosϕ0)−cosαcot−1(acos(α−ϕ0))−asinϕ01+a2{[tan−1tan(α−ϕ0)1+a2+tan−1tanϕ01+a2]}=12{β3−cosαβ2−acosϕ01+a2tanϕ0[tan−1tan(α−ϕ0)1+a2+tan−1tanϕ01+a2]}=12{β3−cosαβ2−[sinβ3−cosαsinβ2cosβ3cosβ2]β1cosβ2sinβ1}

and

(49) $$\begin{array}{cc} \bar{\cos\;\theta }\;A & =\int_{0}^{\alpha }d\phi \int_{0}^{\beta (\phi )}\cos\;\theta \;\sin\;\theta \;d\theta =\frac{1}{2}\int_{0}^{\alpha }\sin^{2}\;\beta (\phi )d\phi =\frac{1}{2}\int_{0}^{\alpha }\frac{d\phi }{1+a^{2}\;\cos^{2}(\phi -\phi_{0})}\\ & =\frac{1∕2}{\sqrt{1+a^{2}}}\left[\tan^{-1}\frac{\tan(\alpha -\phi_{0})}{\sqrt{1+a^{2}}}+\tan^{-1}\frac{\tan\;\phi_{0}}{\sqrt{1+a^{2}}}\right]=\frac{\sin\;\alpha \;\sin\;\beta_{2}\;\sin\;\beta_{3}}{2}\frac{\beta_{1}}{\sin\;\beta_{1}}. \end{array}$$

Let us define the following notations: $t_{x}\equiv \bar{\sin\;\theta \;\cos\;\phi }$, $t_{y}\equiv \bar{\sin\;\theta \;\sin\;\phi }$, and $t_{z}\equiv \bar{\cos\;\theta }$. Since A, $t_{x}\cdot A$, $t_{y}\cdot A$, and $t_{z}\cdot A$ are evaluated exactly in equations (46)–(49) respectively, we have the exact values for *t_x_*, *t_y_*, and *t_z_*. The numerator of our expansion parameter in equation (44) depends on the *θ* and *ϕ* angles:

$$\begin{array}{cc} \hat{\tilde{r}}\cdot [\hat{r}-{\hat{r}}_{\text{ave}}] & =\sin\;\tilde{\theta }\;\cos\;\tilde{\phi }(\sin\;\theta \;\cos\;\phi -t_{x})+\sin\;\tilde{\theta }\;\sin\;\tilde{\phi }(\sin\;\theta \;\sin\;\phi -t_{y})+\cos\;\tilde{\theta }(\cos\;\theta -t_{z})\\ & \equiv \sin\;\tilde{\theta }\;\cos\;\tilde{\phi }({\hat{r}}_{x}-t_{x})+\sin\;\tilde{\theta }\;\sin\;\tilde{\phi }({\hat{r}}_{y}-t_{y})+\cos\;\tilde{\theta }({\hat{r}}_{z}-t_{z}). \end{array}$$

If we keep up to the third term (quadratic in $\hat{\tilde{r}}\cdot [\hat{r}-{\hat{r}}_{\text{ave}}]$), we will need all the following integrals evaluated

(50) q0=∫0αdϕ∫0β(ϕ)sinθdθσ(θ,ϕ)=∫0αdϕ∫0β(ϕ)sinθdθ{σ1+sinθC(ϕ)sinθ+D(ϕ)cosθ[(σ2−σ1)+λ(ϕ)(σ3−σ2)]}=σ1A+∫0αdϕ{[(σ2−σ1)+λ(ϕ)(σ3−σ2)]∫0β(ϕ)sin2θdθC(ϕ)sinθ+D(ϕ)cosθ}=σ1A+∫0αdϕ[(σ2−σ1)+λ(ϕ)(σ3−σ2)][C(1−cosβ)−DsinβC2+D2][+D2(C2+D2)3∕2lnD+(C2+D2+C)tanβ2D−(C2+D2−C)tanβ2].

Here, we can in principle expand the expression to higher order *β* to gain higher order accuracy. However, as we mentioned before, we only keep correction terms of relative order *β*^2^ to the leading term, which itself is of order *β*^2^ for *q*_0_. (This is because the area of the spherical triangle A is proportional to *β*^2^.) Expression (C.1) containing higher order terms are given in appendix C. We thus expand (50) to

(51) $$\begin{array}{cc} q_{0} & =\sigma_{1}A+\int_{0}^{\alpha }d\phi \left[\frac{\sigma_{2}-\sigma_{1}+(\sigma_{3}-\sigma_{2})\lambda (\phi )}{3\;a^{2}\;\cos^{2}(\phi -\phi_{0})}+\frac{\left(10\tilde{h}-31)[(\sigma_{2}-\sigma_{1})+(\sigma_{3}-\sigma_{2})\lambda (\phi )\right]}{120a^{4}\;\cos^{4}(\phi -\phi_{0})}\right]+O(\beta^{6})\\ & \approx \sigma_{1}A+(\sigma_{2}-\sigma_{1})\left[\frac{F(0,0,2)}{3}-\frac{31F(0,0,4)}{120}+\frac{F(1,0,4)}{12}\right]\\ & \;\;+(\sigma_{3}-\sigma_{2})\left[\frac{F(0,1,2)}{3}-\frac{31F(0,1,4)}{120}+\frac{F(1,1,4)}{12}\right]+O(\beta^{6})\\ & \approx \sigma_{1}A+(\sigma_{2}-\sigma_{1})\left[\frac{G(0,0,0,2)}{3}-\frac{31G(0,0,0,4)}{120}+\frac{G(1,0,0,4)}{12}\right]\\ & \;\;+(\sigma_{3}-\sigma_{2})\left[\frac{G(0,1,0,2)+G(0,1,1,2)}{3}-\frac{31G(0,1,0,4)}{120}+\frac{G(1,1,0,4)}{12}\right]+O(\beta^{6}). \end{array}$$

The needed integrals are listed in appendix B.

The next quantity of interest is given by

$$\int_{0}^{\alpha }d\phi \int_{0}^{\beta (\phi )}\sin\;\theta d\theta \;(\sin\;\theta \;\cos\;\phi ,\sin\;\theta \;\sin\;\phi ,\cos\;\theta )\;\sigma (\theta ,\phi ).$$

So in terms of the *θ* integral, we need the following integrals

(52) $$\int_{0}^{\beta (\phi )}\sin^{2}\;\theta d\theta \left\{\sigma_{1}+\frac{\sin\;\theta }{C(\phi )\;\sin\;\theta +D(\phi )\;\cos\;\theta }[(\sigma_{2}-\sigma_{1})+\lambda (\phi )(\sigma_{3}-\sigma_{2})]\right\}$$

(53) $$\int_{0}^{\beta (\phi )}\cos\;\theta \sin\;\theta d\theta \{\sigma_{1}+\frac{\sin\;\theta }{C(\phi )\;\sin\;\theta +D(\phi )\;\cos\;\theta }[(\sigma_{2}-\sigma_{1})+\lambda (\phi )(\sigma_{3}-\sigma_{2})]\}.$$

Both integrals above again can be expanded to higher orders (see appendix C), but we again only keep correction terms of relative order of *β*^2^ to the leading terms. Integral (52) can be evaluated to yield

∫0β(ϕ)sin2θdθ{σ1+sinθC(ϕ)sinθ+D(ϕ)cosθ[(σ2−σ1)+λ(ϕ)(σ3−σ2)]}=σ1(β2−sin(2β)4)+[(σ2−σ1)+λ(ϕ)(σ3−σ2)]{D(cos2β−1)−Csin2β4(C2+D2)}+{Cβ(C2+3D2)2(C2+D2)2−D3(C2+D2)2ln(cosβ+CDsinβ)}≈σ1(β2−sinβcosβ2)+(σ2−σ1)+λ(ϕ)(σ3−σ2)4a3cos3(ϕ−ϕ0){−1+37−6h~30a2cos2(ϕ−ϕ0)}+O(β6),

which upon evaluating $\int_{0}^{\alpha }\;\cos\;\phi d\phi$ yields (with the angular brackets representing average over the charge distribution)

$$\begin{array}{c} \langle {\hat{r}}_{x}\rangle \;q_{0}=\int_{0}^{\alpha }d\phi \int_{0}^{\beta (\phi )}\sin\;\theta d\theta \;(\sin\;\theta \;\cos\;\phi )\;\sigma (\theta ,\phi )\\ \;\;=\sigma_{1}t_{x}A+(\sigma_{2}-\sigma_{1})\left[-\frac{F_{1,0}(0,0,3)}{4}+\frac{37F_{1,0}(0,0,5)}{120}-\frac{F_{1,0}(1,0,5)}{20}\right]\\ \;\;\;+(\sigma_{3}-\sigma_{2})\left[-\frac{F_{1,0}(0,1,3)}{4}+\frac{37F_{1,0}(0,1,5)}{120}-\frac{F_{1,0}(1,1,5)}{20}\right]+O(\beta^{7})\\ \;\;=\sigma_{1}t_{x}A+(\sigma_{2}-\sigma_{1})\left[-\frac{G_{1,0}(0,0,0,3)}{4}+\frac{37G_{1,0}(0,0,0,5)}{120}-\frac{G_{1,0}(1,0,0,5)}{20}\right]\\ \;\;\;+(\sigma_{3}-\sigma_{2})\left[-\frac{-G_{1,0}(0,1,0,3)+G_{1,0}(0,1,1,3)}{4}+\frac{37G_{1,0}(0,1,0,5)}{120}-\frac{G_{1,0}(1,1,0,5)}{20}\right]+O(\beta^{7}). \end{array}$$

Again, the integrals needed are provided in appendix B. Similar integration over $\int_{0}^{\alpha }\;\sin\;\phi d\phi$ yields

$$\begin{array}{cc} \langle {\hat{r}}_{y}\rangle \;q_{0} & =\int_{0}^{\alpha }d\phi \int_{0}^{\beta (\phi )}\sin\;\theta d\theta \;(\sin\;\theta \;\sin\;\phi )\;\sigma (\theta ,\phi )\\ & =\sigma_{1}t_{y}A+(\sigma_{2}-\sigma_{1})\left[-\frac{F_{0,1}(0,0,3)}{4}+\frac{37F_{0,1}(0,0,5)}{120}-\frac{F_{0,1}(1,0,5)}{20}\right]\\ & \;\;+(\sigma_{3}-\sigma_{2})\left[-\frac{F_{0,1}(0,1,3)}{4}+\frac{37F_{0,1}(0,1,5)}{120}-\frac{F_{0,1}(1,1,5)}{20}\right]+O(\beta^{7})\\ & =\sigma_{1}t_{y}A+(\sigma_{2}-\sigma_{1})\left[-\frac{G_{0,1}(0,0,0,3)}{4}+\frac{37G_{0,1}(0,0,0,5)}{120}-\frac{G_{0,1}(1,0,0,5)}{20}\right]\\ & \;\;+(\sigma_{3}-\sigma_{2})\left[-\frac{-G_{0,1}(0,1,0,3)+G_{0,1}(0,1,1,3)}{4}+\frac{37G_{0,1}(0,1,0,5)}{120}-\frac{G_{0,1}(1,1,0,5)}{20}\right]+O(\beta^{7}). \end{array}$$

The integrals needed are provided in appendix B. The integral (53) can also be evaluated to yield

∫0β(ϕ)cosθsinθdθ{σ1+sinθC(ϕ)sinθ+D(ϕ)cosθ[(σ2−σ1)+λ(ϕ)(σ3−σ2)]}=σ1sin2β2+[(σ2−σ1)+λ(ϕ)(σ3−σ2)]{C(1−cos2β)−Dsin2β4(C2+D2)}−{Dβ(C2−D2)2(C2+D2)2+CD2(C2+D2)2ln(cosβ+CDsinβ)}≈σ1sin2β2+(σ2−σ1)+λ(ϕ)(σ3−σ2)3a2cos2(ϕ−ϕ0)[1+10h~−4340a2cos2(ϕ−ϕ0)}+O(β6)

which upon evaluating $\int_{0}^{\alpha }d\phi$ yields

$$\begin{array}{cc} \langle {\hat{r}}_{z}\rangle \;q_{0} & =\int_{0}^{\alpha }d\phi \int_{0}^{\beta (\phi )}\sin\;\theta d\theta \;(\cos\;\theta )\;\sigma (\theta ,\phi )\\ & \approx \sigma_{1}t_{z}A+(\sigma_{2}-\sigma_{1})\left[\frac{F(0,0,2)}{3}-\frac{43F(0,0,4)}{120}+\frac{F(1,0,4)}{12}\right]\\ & \;\;+(\sigma_{3}-\sigma_{2})\left[\frac{F(0,1,2)}{3}-\frac{43F(0,1,4)}{120}+\frac{F(1,1,4)}{12}\right]+O(\beta^{6})\\ & \approx \sigma_{1}t_{z}A+(\sigma_{2}-\sigma_{1})\left[\frac{G(0,0,0,2)}{3}-\frac{43\;G(0,0,0,4)}{120}+\frac{G(1,0,0,4)}{12}\right]\\ & \;\;+(\sigma_{3}-\sigma_{2})\left[\frac{G(0,1,0,2)+G(0,1,1,2)}{3}-\frac{43\;G(0,1,0,4)}{120}+\frac{G(1,1,0,4)}{12}\right]+O(\beta^{6}). \end{array}$$

The integrals needed are provided in appendix B.

For the same reason, to compute the average (in the presence of charge distribution) of ${\left(\hat{\tilde{r}}\cdot [\hat{r}-\langle \hat{r}\rangle ]\right)}^{2}$, the following integrals will be encountered

(54) $$\int_{0}^{\beta (\phi )}\sin^{3}\;\theta d\theta \left\{\sigma_{1}+\frac{\sin\;\theta }{C(\phi )\;\sin\;\theta +D(\phi )\;\cos\;\theta }[(\sigma_{2}-\sigma_{1})+\lambda (\phi )(\sigma_{3}-\sigma_{2})]\right\}$$

(55) $$\int_{0}^{\beta (\phi )}\cos\;\theta \;\sin^{2}\;\theta d\theta \left\{\sigma_{1}+\frac{\sin\;\theta }{C(\phi )\;\sin\;\theta +D(\phi )\;\cos\;\theta }[(\sigma_{2}-\sigma_{1})+\lambda (\phi )(\sigma_{3}-\sigma_{2})]\right\}$$

(56) $$\int_{0}^{\beta (\phi )}\cos^{2}\;\theta \;\sin\;\theta d\theta \left\{\sigma_{1}+\frac{\sin\;\theta }{C(\phi )\;\sin\;\theta +D(\phi )\;\cos\;\theta }[(\sigma_{2}-\sigma_{1})+\lambda (\phi )(\sigma_{3}-\sigma_{2})]\right\}.$$

Equation (54) can be integrated to yield

(57) σ1[(1−cosβ)−13(1−cos3β)]+[(σ2−σ1)+λ(ϕ)(σ3−σ2)]{C(cos3β−1)−Dsin3β3(C2+D2)−C(C2+2D2)(cosβ−1)+D3sinβ(C2+D2)2{+D4(C2+D2)5∕2lnD+(C2+D2+C)tanβ2D−(C2+D2−C)tanβ2}≈σ1[(1−cosβ)−13(1−cos3β)]+(σ2−σ1)+λ(ϕ)(σ3−σ2)5a4cos4(ϕ−ϕ0)[1+14h~−14384a2cos2(ϕ−ϕ0)]+O(β9).

Consequently, if we multiply equation (54) by cos^2^ *ϕ* and integrate over *ϕ* from 0 to *α*, we obtain

(58) 〈r^x2〉q0=∫0αdϕ∫0β(ϕ)sinθdθ(sin2θcos2ϕ)σ(θ,ϕ)≈∫0ασ1[(1−cosβ(ϕ))−13(1−cos3β(ϕ))]cos2ϕdϕ+∫0α(σ2−σ1)+λ(ϕ)(σ3−σ2)5a4cos4(ϕ−ϕ0)[1+14h~−14384a2cos2(ϕ−ϕ0)]cos2ϕdϕ+O(β9)≈σ13{A+sinαcosα+a3cos2ϕ0sinϕ0(1+a2)1+a2cos2ϕ0−cos2αasin(α−ϕ0)(1+a2)1+a2cos2(α−ϕ0)}{−1+a2cos2(α−ϕ0)(1+a2)asin(α+ϕ0)}+σ2−σ15F2,0(0,0,4)+σ3−σ25F2,0(0,1,4)+O(β7)≈σ13{A+sinαcosα+a3cos2ϕ0sinϕ0(1+a2)1+a2cos2ϕ0−cos2αasin(α−ϕ0)(1+a2)1+a2cos2(α−ϕ0){−1+a2cos2(α−ϕ0)(1+a2)[sin(2α)acos(α−ϕ0)−cos2αasin(α−ϕ0)]}+σ3−σ25G2,0(0,1,0,4)+σ2−σ15G2,0(0,0,0,4)+O(β7).

If we multiply equation (54) by cos *ϕ* sin *ϕ* and integrate over *ϕ* from 0 to *α*, we obtain

(59) 〈r^xr^y〉q0=∫0αdϕ∫0β(ϕ)sinθdθ(sin2θcosϕ,sinϕ)σ(θ,ϕ)≈∫0ασ1[(1−cosβ(ϕ))−13(1−cos3β(ϕ))]cosϕsinϕdϕ+∫0α(σ2−σ1)+λ(ϕ)(σ3−σ2)5a4cos4(ϕ−ϕ0)[1+14h~−14384a2cos2(ϕ−ϕ0)]cosϕsinϕdϕ+O(β9)≈σ13{sin2α−1+a2cos2ϕ01+a2acosϕ0−sinαcosαasin(α−ϕ0)(1+a2)1+a2cos2(α−ϕ0)}+{1+a2cos2(α−ϕ0)1+a2acos(α+ϕ0)}+σ2−σ15F1,1(0,0,4)+σ3−σ25F1,1(0,1,4)+O(β7)≈σ13{sin2α−1+a2cos2ϕ01+a2acosϕ0−sinαcosαasin(α−ϕ0)(1+a2)1+a2cos2(α−ϕ0)}+{1+a2cos2(α−ϕ0)1+a2[cos(2α)acos(α−ϕ0)+sin(2α)asin(α−ϕ0)]}+σ3−σ25G1,1(0,1,0,4)+σ2−σ15G1,1(0,0,0,4)+O(β7).

And similarly, if we multiply equation (54) by sin^2^ *ϕ* and integrate over *ϕ* from 0 to *α*, we obtain

(60) 〈r^y2〉q0=∫0αdϕ∫0β(ϕ)sinθdθ(sin2θsin2ϕ)σ(θ,ϕ)≈∫0ασ1[(1−cosβ(ϕ))−13(1−cos3β(ϕ))]sin2ϕdϕ+∫0α(σ2−σ1)+λ(ϕ)(σ3−σ2)5a4cos4(ϕ−ϕ0)[1+14h~−14384a2cos2(ϕ−ϕ0)]sin2ϕdϕ+O(β9)≈σ13{A−sinαcosα−1+a2cos2ϕ0(1+a2)asinϕ0−sin2αasin(α−ϕ0)(1+a2)1+a2cos2(α−ϕ0)}+{1+a2cos2(α−ϕ0)(1+a2)asin(α+ϕ0)}+σ2−σ15F0,2(0,0,4)+σ3−σ25F0,2(0,1,4)+O(β7)≈σ13{A−sinαcosα−1+a2cos2ϕ0(1+a2)asinϕ0−sin2αasin(α−ϕ0)(1+a2)1+a2cos2(α−ϕ0)}+{1+a2cos2(α−ϕ0)(1+a2)[sin(2α)acos(α−ϕ0)−cos2αasin(α−ϕ0)]}+σ3−σ25G0,2(0,1,0,4)+σ2−σ15G0,2(0,0,0,4)+O(β7).

Equation (55) can be integrated to yield

(61) σ1sin3β3+[(σ2−σ1)+λ(ϕ)(σ3−σ2)]{D(cos3β−1)+Csin3β3(C2+D2)}+D2{D(1−cosβ)+Csinβ(C2+D2)2−CD3(C2+D2)5∕2lnD+(C2+D2+C)tanβ2D−(C2+D2−C)tanβ2}≈σ1sin3β3+(σ2−σ1)+λ(ϕ)(σ3−σ2)4a3cos3(ϕ−ϕ0)[1+6h~−4730a2cos2(ϕ−ϕ0)][+3377−456h~+280h~21680a4cos4(ϕ−ϕ0)]+O(β10).

If we multiply the integral above by cos *ϕ* and integrate over *ϕ* from 0 to *α*, we obtain

(62) $$\begin{array}{cc} \langle {\hat{r}}_{x}{\hat{r}}_{z}\rangle \;q_{0} & =\int_{0}^{\alpha }d\phi \int_{0}^{\beta (\phi )}\sin\;\theta d\theta \;(\sin\;\theta \;\cos\;\phi \;\cos\;\theta )\;\sigma (\theta ,\phi )\\ & \approx \int_{0}^{\alpha }\sigma_{1}\frac{\sin^{3}\beta (\phi )}{3}\cos\;\phi \;d\phi \\ & \;\;+\int_{0}^{\alpha }\cos\;\phi \;d\;\phi \frac{\left(\sigma_{2}-\sigma_{1})+\lambda (\phi )(\sigma_{3}-\sigma_{2}\right)}{4\;a^{3}\cos^{3}(\phi -\phi_{0})}\left[1+\frac{6\tilde{h}-47}{30\;a^{2}\cos^{2}(\phi -\phi_{0})}\right]+O(\beta^{8})\\ & \approx \frac{\sigma_{1}}{3}\left\{\frac{\sin\;\alpha +a^{2}\;\cos(\alpha -\phi_{0})\sin\;\phi_{0}}{(1+a^{2})\sqrt{1+a^{2}\;\cos^{2}(\alpha -\phi_{0})}}-\frac{a^{2}\;\cos\;\phi_{0}\;\sin\;\phi_{0}}{(1+a^{2})\sqrt{1+a^{2}\cos^{2}\;\phi_{0}}}\right\}\\ & \;+\frac{\sigma_{2}-\sigma_{1}}{4}\left[F_{1,0}(0,0,3)+\frac{F_{1,0}(1,0,5)}{5}-\frac{47F_{1,0}(0,0,5)}{30}\right]\\ & \;+\frac{\sigma_{3}-\sigma_{2}}{4}\left[F_{1,0}(0,1,3)+\frac{F_{1,0}(1,1,5)}{5}-\frac{47F_{1,0}(0,1,5)}{30}\right]+O(\beta^{8})\\ & \approx \frac{\sigma_{1}}{3}\left\{\frac{\sin\;\alpha +a^{2}\cos(\alpha -\phi_{0})\sin\;\phi_{0}}{(1+a^{2})\sqrt{1+a^{2}\;\cos^{2}(\alpha -\phi_{0})}}-\frac{a^{2}\;\cos\;\phi_{0}\;\sin\;\phi_{0}}{(1+a^{2})\sqrt{1+a^{2}\;\cos^{2}\;\phi_{0}}}\right\}\\ & \;+\frac{\sigma_{3}-\sigma_{2}}{4}\left[G_{1,0}(0,1,0,3)+G_{1,0}(0,1,1,3)+\frac{G_{1,0}(1,1,0,5)}{5}-\frac{47G_{1,0}(0,1,0,5)}{30}\right]\\ & \;+\frac{\sigma_{2}-\sigma_{1}}{4}\left[G_{1,0}(0,0,0,3)+\frac{G_{1,0}(1,0,0,5)}{5}-\frac{47G_{1,0}(0,0,0,5)}{30}\right]+O(\beta^{8}). \end{array}$$

Similarly, if we multiply (61) by sin *ϕ* and integrate over *ϕ*, we obtain

(63) $$\begin{array}{cc} \langle {\hat{r}}_{y}{\hat{r}}_{z}\rangle \;q_{0} & =\int_{0}^{\alpha }d\phi \int_{0}^{\beta (\phi )}\sin\;\theta d\theta \;(\sin\;\theta \;\sin\;\phi \;\cos\;\theta )\;\sigma (\theta ,\phi )\\ & \approx \int_{0}^{\alpha }\sigma_{1}\frac{\sin^{3}\beta (\phi )}{3}\sin\;\phi \;d\phi \\ & \;\;+\int_{0}^{\alpha }\sin\;\phi \;d\;\phi \frac{\left(\sigma_{2}-\sigma_{1})+\lambda (\phi )(\sigma_{3}-\sigma_{2}\right)}{4a^{3}\cos^{3}(\phi -\phi_{0})}\left[1+\frac{6\tilde{h}-47}{30a^{2}\cos^{2}(\phi -\phi_{0})}\right]+O(\beta^{8})\\ & \approx \frac{\sigma_{1}}{3}\left\{\frac{\sqrt{1+a^{2}\cos^{2}\phi_{0}}}{\left(1+a^{2}\right)}-\frac{\cos\;\alpha +a^{2}\cos(\alpha -\phi_{0})\cos\;\phi_{0}}{(1+a^{2})\sqrt{1+a^{2}\cos^{2}(\alpha -\phi_{0})}}\right\}\\ & \;+\frac{\sigma_{2}-\sigma_{1}}{4}\left[F_{0,1}(0,0,3)+\frac{F_{0,1}(1,0,5)}{5}-\frac{47F_{0,1}(0,0,5)}{30}\right]\\ & \;+\frac{\sigma_{3}-\sigma_{2}}{4}\left[F_{0,1}(0,1,3)+\frac{F_{0,1}(1,1,5)}{5}-\frac{47F_{0,1}(0,1,5)}{30}\right]+O(\beta^{8})\\ & \approx \frac{\sigma_{1}}{3}\left\{\frac{\sqrt{1+a^{2}\cos^{2}\phi_{0}}}{\left(1+a^{2}\right)}-\frac{\cos\;\alpha +a^{2}\cos(\alpha -\phi_{0})\cos\;\phi_{0}}{(1+a^{2})\sqrt{1+a^{2}\cos^{2}(\alpha -\phi_{0})}}\right\}\\ & \;+\frac{\sigma_{3}-\sigma_{2}}{4}\left[G_{0,1}(0,1,0,3)+G_{0,1}(0,1,1,3)+\frac{G_{0,1}(1,1,0,5)}{5}-\frac{47G_{0,1}(0,1,0,5)}{30}\right]\\ & \;+\frac{\sigma_{2}-\sigma_{1}}{4}\left[G_{0,1}(0,0,0,3)+\frac{G_{0,1}(1,0,0,5)}{5}-\frac{47G_{0,1}(0,0,0,5)}{30}\right]+O(\beta^{8}). \end{array}$$

Equation (56) can be integrated to yield

(64) σ11−cos3β3+[(σ2−σ1)+λ(ϕ)(σ3−σ2)]{C(1−cos3β)+Dsin3β3(C2+D2)}{−CDD(1−cosβ)+Csinβ(C2+D2)2+C2D2(C2+D2)5∕2lnD+(C2+D2+C)tanβ2D−(C2+D2−C)tanβ2}≈σ11−cos3β3+(σ2−σ1)+λ(ϕ)(σ3−σ2)3a2cos2(ϕ−ϕ0)[1+2h~−118a2cos2(ϕ−ϕ0)+[269−44h~+36h~2160a4cos4(ϕ−ϕ0)]+O(β9).

If we integrate (64) over the *ϕ* angle, we obtain

(65) $$\begin{array}{cc} \langle {\hat{r}}_{z}^{2}\rangle \;q_{0} & =\int_{0}^{\alpha }d\phi \int_{0}^{\beta (\phi )}\sin\;\theta d\theta \;(\cos^{2}\;\theta )\;\sigma (\theta ,\phi )\\ & \approx \int_{0}^{\alpha }\sigma_{1}\frac{1-\cos^{3}\beta (\phi )}{3}d\phi +\int_{0}^{\alpha }d\phi \frac{\left(\sigma_{2}-\sigma_{1})+\lambda (\phi )(\sigma_{3}-\sigma_{2}\right)}{3\;a^{2}\cos^{2}(\phi -\phi_{0})}\\ & \;\times \left[1+\frac{2\tilde{h}-11}{8\;a^{2}\cos^{2}(\phi -\phi_{0})}+\frac{269-44\tilde{h}+36\overset{\sim^{2}}{h}}{160\;a^{4}\cos^{4}(\phi -\phi_{0})}\right]+O(\beta^{9})\\ & \approx \frac{\sigma_{1}}{3}\left\{A+\frac{a\;\sin(\alpha -\phi_{0})}{(1+a^{2})\sqrt{1+a^{2}\cos^{2}(\alpha -\phi_{0})}}+\frac{a\;\sin\;\phi_{0}}{(1+a^{2})\sqrt{1+a^{2}\cos^{2}\phi_{0}}}\right\}\\ & \;+\frac{\sigma_{2}-\sigma_{1}}{3}\left[F(0,0,2)+\frac{F(1,0,4)}{4}-\frac{11F(0,0,4)}{8}\right]\\ & \;+\frac{\sigma_{3}-\sigma_{2}}{3}\left[F(0,1,2)+\frac{F(1,1,4)}{4}-\frac{11F(0,1,4)}{8}\right]+O(\beta^{7})\\ & \approx \frac{\sigma_{1}}{3}\left\{A+\frac{a\;\sin(\alpha -\phi_{0})}{(1+a^{2})\sqrt{1+a^{2}\cos^{2}(\alpha -\phi_{0})}}+\frac{a\;\sin\phi_{0}}{(1+a^{2})\sqrt{1+a^{2}\cos^{2}\phi_{0}}}\right\}\\ & \;+\frac{\sigma_{3}-\sigma_{2}}{3}\left[G(0,1,0,2)+G(0,1,1,2)-\frac{11G(0,1,0,4)}{8}+\frac{G(1,1,0,4)}{4}\right]\\ & \;+\frac{\sigma_{2}-\sigma_{1}}{3}\left[G(0,0,0,2)-\frac{11G(0,0,0,4)}{8}+\frac{G(1,0,0,4)}{4}\right]+O(\beta^{7}). \end{array}$$

All integrals needed are shown in appendix B.

### 3.6. Electric field outside the sphere

The electric potential produced by the spherical triangle (with 0 ⩽ *ϕ* ⩽ *α* and 0 ⩽ *θ* ⩽ tan^−1^ $\left(\frac{1}{a\;\cos(\phi -\phi_{0})}\right)$) at a location $\tilde{r}$ outside the sphere can be written as

$$\int_{0}^{\alpha }d\phi \int_{0}^{\beta (\phi )}\sin\;\theta d\theta \frac{\sigma (\theta ,\phi )}{|\tilde{r}-r|}R^{2}=\sum_{l=0}^{\infty }\frac{r^{l}}{{\tilde{r}}^{l+1}}\frac{4\pi R^{2}}{2l+1}\sum_{m=-l}^{l}Y_{lm}(\tilde{\theta },\tilde{\phi })\int_{0}^{\alpha }d\phi \int_{0}^{\beta (\phi )}\sin\;\theta d\theta \;Y_{lm}^{*}(\theta ,\phi )\sigma (\theta ,\phi ).$$

This expansion, however, converges fast only if $\tilde{r}\gg r$. When $|\tilde{r}-r|$ is too small, the only way to get accurate potential and electric field contributions is through numerical integration. However, there are cases when $\tilde{r}$ is not too much larger than *r* and we can expand the integral in such a way that it still converges reasonably fast. The idea is a straightforward extension from the previous section. We write

(66) 1∣r~−r∣=1∣(r~−rave)−(r−rave)∣=1∣r~−rave∣[1−2(r~−rave)⋅(r−rave)−(r−rave)⋅(r−rave)∣r~−rave∣2]−1∕2=1∣r~−rave∣[1−2r~⋅(r−rave)+rave2−r2∣r~−rave∣2]−1∕2=1∣r~−rave∣[1−2r~⋅(r−rave)+rave2−r22∣r~−rave∣2+38(2r~⋅(r−rave)+rave2−r2∣r~−rave∣2)2][+516(2r~⋅(r−rave)+rave2−r2∣r~−rave∣2)3+⋯].

The calculations shown in the previous section and their extensions provide the formulas needed and we thus will not repeat them here.

## 4. Discussions and conclusions

The reader might have noticed that we did not discuss the possible singularities [13, 14] that may arise from the molecular surface definition of Richards and Connolly. This is because we are mainly interested in applying the classical formalism here to the explicit solvent model where we are allowed to make the probe sphere (whose sole functionality is to provide a smooth surface for applying boundary condition) small enough to avoid these potential singularities.

In this manuscript, we lay the first part of the ground work for employing curved patches for applying surface charge method in electrostatics. We have analytically shown how one may control the accuracy by expanding in powers of the arc length (multiplied by the curvature). Before we conclude this paper, let us illustrate with the computation of the area of a spherical triangle using the series expansion method outlined here versus the exact answer. Let us consider a spherical triangle with one vertex on the north pole, (0, 0, 1), the second vertex having coordinates (sin *β*, 0, cos *β*) and the third vertex (sin *β* cos *α*, sin *β* sin *α*, cos *β*) with *β* ≪ 1 and *α* = *π*/3. We then have *ϕ*_0_ = *π*/6 and $a=\frac{2}{\sqrt{3}}\cot\;\beta$. We have evaluated the area exactly in equation (46) and in this case

$$A=\frac{\pi }{3}-2\;\tan^{-1}\frac{\cos\;\beta }{\sqrt{3}}.$$

Using (20) we may obtain the series expression that we use

$$\begin{array}{cc} A & =\int_{0}^{\alpha }\int_{0}^{\beta (\phi )}\sin\;\theta \;d\theta \;d\phi =\int_{0}^{\alpha }(1-\cos\;\beta (\phi ))d\phi \\ & =\int_{0}^{\alpha }\left\{\frac{1}{2[a^{2}\;\cos^{2}(\phi -\phi_{0})]}-\frac{3}{8[a^{2}\;\cos^{2}(\phi -\phi_{0}){]}^{2}}+\frac{5}{16[a^{2}\;\cos^{2}(\phi -\phi_{0}){]}^{3}}+\cdots \right\}d\phi \\ & =\frac{F(0,0,2)}{2}+\frac{3F(0,0,4)}{8}+\frac{5F(0,0,6)}{16}+\cdots \end{array}$$

When *β* = 0.15, the exact answer yields the numerical value 0.009 751 83; the first two terms $\frac{F(0,0,2)}{2}+\frac{3F(0,0,4)}{8}$ yield the numerical value 0.009 749 61 while including the $\frac{5F(0,0,6)}{16}$ term makes the series value 0.009 751 87. On the other hand, if we choose *β* = 0.1, the numerical value from the exact answer becomes 0.004 331 92; the first two terms $\frac{F(0,0,2)}{2}+\frac{3F(0,0,4)}{8}$ yield the numerical value 0.004 331 73 while including the $\frac{5F(0,0,6)}{16}$ term makes the series value 0.004 331 92. Evidently, the smaller the triangular patches are, the better our approximation becomes. For reference, we have provided enough details for obtaining higher order corrections.

In the forthcoming manuscript, we shall cover the other type of surface, the toroid, along with a proper way to deal with triangles with one side not belonging to a great arc, which occur at the jointing region where spherical surface and toroidal surface meet. In addition, we will provide a discussion on the disappearance of divergence of the electric field when curved surface patches, developed in this paper and the forthcoming paper, are employed. We will also, in the third paper of the series, apply the fundamental work developed here and in the forthcoming paper to investigate molecular interaction at short distance. In this case, because the electric field experienced by a molecule may vary significantly from one of its atoms to the others, approximating the molecule as a single dielectric sphere with multipole moments may be insufficient; using the more general molecular shape, composed of partial toroids and spheres, may be better suited to deal with cases where external electric field has non-negligible variation across a molecule.

## Appendix A. Geodesic on a sphere

For the sake of completeness, we provide in this appendix the derivation of equation (1). Points on a sphere can can be parametrized by the polar angle *θ* and the azimuthal angle *ϕ*. On a sphere, the distance between two infinitesimally separated points (*θ*, *ϕ*) and (*θ* + d*θ*, *ϕ* + d*ϕ*) is given by

$$ds^{2}=(\sin\;\theta \;d\phi {)}^{2}+(d\theta {)}^{2}.$$

An arbitrary path connecting two points *A* and *B* can be divided into infinitely many infinitesimal segments, leading to path length

$$S=\int_{\theta_{A}}^{\theta_{B}}\sqrt{\sin^{2}\;\theta {\left(\frac{\partial \phi }{\partial \theta }\right)}^{2}+1}\;d\theta \equiv \int_{\theta_{A}}^{\theta_{B}}L(\phi ,\dot{\phi })\;d\theta ,$$

where $\dot{\phi }\equiv \partial \phi ∕\partial \theta$. Extremizing the distance between points *A* and *B* on the sphere leads to

(A.1) $$0=\frac{d}{d\theta }\frac{\partial L}{\partial \dot{\phi }}-\frac{\partial L}{\partial \phi }.$$

Since L is independent of *ϕ*, ∂L∕∂ϕ=0 and $\frac{\partial L}{\partial \phi }$ becomes a constant

(A.2) $$c=\frac{\partial L}{\partial \dot{\phi }}=\frac{\sin^{2}\;\theta \;\frac{\partial \phi }{\partial \phi }}{\sqrt{\sin^{2}\;\theta {\left(\frac{\partial \phi }{\partial \theta }\right)}^{2}+1}}.$$

Note that ∣*c*∣ < 1 because

$$|c|=\frac{|\sin\;\theta \;\frac{\partial \phi }{\partial \phi }|}{\sqrt{|\sin\;\theta \frac{\partial \phi }{\partial \theta }{|}^{2}+1}}|\sin\;\theta |<|\sin\;\theta |⩽1.$$

Squaring both sides of equation (A.2) and with some algebra, we obtain

(A.3) $${\left(\frac{\partial \phi }{\partial \theta }\right)}^{2}=\frac{c^{2}}{\sin^{4}\;\theta \;(1-c^{2}∕\sin^{2}\;\theta )}$$

or

(A.4) $$\frac{\partial \phi }{\partial \theta }=\frac{\pm |c|}{\sin^{2}\;\theta \sqrt{1-\frac{c^{2}}{\sin^{2}\;\theta }}}\Rightarrow \;\phi =\int \frac{\pm |c|}{\sin^{2}\;\theta \sqrt{1-\frac{c^{2}}{\sin^{2}\;\theta }}}d\theta .$$

Let *u* ≡ cot *θ*; then $du=-\frac{d\theta }{\sin^{2}\;\theta }$ and the integral above becomes (with $a=\frac{\sqrt{1-c^{2}}}{|c|}$)

$$\begin{array}{cc} \phi & =\int \frac{\mp |c|du}{\sqrt{1-c^{2}(u^{2}+1)}}=\mp \int \frac{du}{\sqrt{\frac{1-c^{2}}{c^{2}}-u^{2}}}=\mp \int \frac{du}{\sqrt{a^{2}-u^{2}}}\\ & =\mp \;\cos^{-1}(\frac{u}{a})+\phi_{0}, \end{array}$$

implying

(A.5) $$\cos(\phi -\phi_{0})=\frac{u}{a}=\frac{\cot\;\theta }{a}$$

or

(A.6) $$\cot\;\theta =a\;\cos(\phi -\phi_{0})$$

exactly equation (1).

## Appendix B. List of some integrals from equation (36)

$$\begin{array}{cc} G(0,0,0,1)= & \frac{1}{2a}\text{ln}\frac{\left[1+\sin(\alpha -\phi_{0})][1+\sin(\phi_{0})\right]}{\left[1-\sin(\alpha -\phi_{0})[1-\sin(\phi_{0})\right]}\\ G(0,0,0,3)= & \frac{1}{4a^{3}}\text{ln}\frac{\left[1+\sin(\alpha -\phi_{0})][1+\sin(\phi_{0})\right]}{\left[1-\sin(\alpha -\phi_{0})][1-\sin(\phi_{0})\right]}+\frac{1}{2a^{3}}\left(\frac{\sin(\alpha -\phi_{0})}{\cos^{2}(\alpha -\phi_{0})}+\frac{\sin\;\phi_{0}}{\cos^{2}\;\phi_{0}}\right)\\ G(0,0,0,5)= & \frac{3}{16a^{5}}\text{ln}\frac{\left[1+\sin(\alpha -\phi_{0})][1+\sin(\phi_{0})\right]}{\left[1-\sin(\alpha -\phi_{0})][1-\sin(\phi_{0})\right]}+\frac{3}{8a^{5}}\left(\frac{\sin(\alpha -\phi_{0})}{\cos^{2}(\alpha -\phi_{0})}+\frac{\sin\;\phi_{0}}{\cos^{2}\;\phi_{0}}\right)\\ & +\frac{1}{4a^{5}}\left(\frac{\sin(\alpha -\phi_{0})}{\cos^{4}(\alpha -\phi_{0})}+\frac{\sin\;\phi_{0}}{\cos^{4}\;\phi_{0}}\right) \end{array}$$

$$\begin{array}{cc} G(1,0,0,3)= & -\frac{\zeta_{23}[\cos\;\alpha -3\;\cos(\alpha -2\phi_{0})]\cos^{2}\;\phi_{0}\;\sin\;\beta_{3}}{8a\;\sin^{2}\;\alpha \;\sin\;\beta_{2}}\text{ln}\frac{\left[1+\sin(\alpha -\phi_{0})][1+\sin(\phi_{0})\right]}{\left[1-\sin(\alpha -\phi_{0})][1-\sin(\phi_{0})\right]}\\ & -\frac{\zeta_{23}\cos\;\beta_{3}}{2a\;\sin^{2},\alpha \;\cos\;\beta_{2}}[2(\sin\;\phi_{0}+\sin(\alpha -\phi_{0})\;\cos\;\phi_{0}\;\cos(\alpha -\phi_{0})\\ & -\sin(\alpha -\phi_{0})\;\cos^{2}\;\phi_{0}-\sin\;\phi_{0}\;\cos^{2}(\alpha -\phi_{0})] \end{array}$$

$$\begin{array}{cc} G(0,1,0,1)= & \frac{\sin\;\beta_{3}\;\sin(2\phi_{0})}{4a\;\sin\;\alpha \;\sin\;\beta_{2}}\text{ln}\frac{\left[1+\sin(\alpha -\phi_{0})][1+\sin(\phi_{0})\right]}{\left[1-\sin(\alpha -\phi_{0})][1-\sin(\phi_{0})\right]}\\ & +\frac{[\cos\;\phi_{0}-\cos(\alpha -\phi_{0})]\cos\;\beta_{3}}{a\;\sin\;\alpha \;\cos\;\beta_{2}} \end{array}$$

$$\begin{array}{cc} G(0,1,1,1)= & [2-3\;\cos^{2}\;\phi_{0}]\frac{\sin^{2}\;\beta_{3}\;(\cos\;\beta_{2}-\cos\;\beta_{3})\;\cos^{2}\;\phi_{0}}{4a\;\sin^{2}\;\alpha \;\cos\;\beta_{3}\;\sin^{2}\;\beta_{2}}\text{ln}\frac{\left[1+\sin(\alpha -\phi_{0})][1+\sin(\phi_{0})\right]}{\left[1-\sin(\alpha -\phi_{0})][1-\sin(\phi_{0})\right]}\\ & +\frac{(\cos,\beta_{2}-\cos\;\beta_{3})\;\cos\;\beta_{3}}{2a\;\sin^{2}\;\alpha \;\cos^{2}\;\beta_{2}}[2\;\sin(\alpha -\phi_{0})\;\cos^{2}\;\phi_{0}+8\;\sin\;\phi_{0}\;\cos\;\phi_{0}\;\cos(\alpha -\phi_{0})\\ & -6\;\sin\;\phi_{0}\;\cos^{2}(\alpha -\phi_{0})] \end{array}$$

$$\begin{array}{cc} G(0,1,0,3)= & \frac{\sin\;\beta_{3}\;\sin\;\phi_{0}\;\cos\;\phi_{0}}{4a^{3}\;\sin\;\alpha \;\sin\;\beta_{2}}\text{ln}\frac{\left[1+\sin(\alpha -\phi_{0})][1+\sin(\phi_{0})\right]}{\left[1-\sin(\alpha -\phi_{0})][1-\sin(\phi_{0})\right]}\\ & +\frac{\cos\;\beta_{3}}{6a^{3}\;\sin\;\alpha \;\cos\;\beta_{2}\;\cos^{2}\;\phi_{0}\;\cos^{2}(\alpha -\phi_{0})}[\cos^{3}(\alpha -\phi_{0})(1-3\;\cos^{2}\;\phi_{0})\\ & +2\;\cos^{3}\;\phi_{0}+3\;\sin\;\phi_{0}\;\sin(\alpha -\phi_{0})\;\cos^{2}\;\phi_{0}\;\cos(\alpha -\phi_{0})] \end{array}$$

$$\begin{array}{cc} G(1,1,0,3)= & \frac{\zeta_{23}\;\sin^{2}\;\beta_{3}\;\cos^{3}\;\phi_{0}}{16a\;\sin^{3}\;\alpha \;\sin^{2}\;\beta_{2}}\text{ln}\frac{\left[1+\sin(\alpha -\phi_{0})][1+\sin(\phi_{0})\right]}{\left[1-\sin(\alpha -\phi_{0})][1-\sin(\phi_{0})\right]}\\ & \times \left[\sin\;\alpha \;\cos\;\theta_{0}+(8-13\;\cos^{2}\;\theta_{0})\;\sin(\alpha -\phi_{0})+7\;\cos(\alpha -\phi_{0})\;\cos\;\phi_{0}\;\sin\;\phi_{0}\right]\\ & +\frac{\zeta_{23}\;\cos^{2}\;\beta_{3}}{6a\;\sin^{3}\;\alpha \;\cos^{2}\;\beta_{2}}\{2\;\cos^{3}(\alpha -\phi_{0})-6\;\cos^{2}(\alpha -\phi_{0})\;\cos\;\phi_{0}\\ & +3\;\sin\;\phi_{0}\;\sin(\alpha -\phi_{0})[2\;\cos(\alpha -\phi_{0})\;\cos^{2}\;\phi_{0}-3\;\cos^{2}(\alpha -\phi_{0})\;\cos\;\phi_{0}]\\ & -3\;\cos(\alpha -\phi_{0})\;\cos\;\phi_{0}[2\;\cos^{2}(\alpha -\phi_{0})\;\cos\;\phi_{0}-3\;\cos(\alpha -\phi_{0})\;\cos^{2}\;\phi_{0}]\} \end{array}$$

$$\begin{array}{cc} G(0,0,0,2) & =\frac{\sin\;\alpha }{a^{2}\;\cos\;\phi_{0}\;\cos(\alpha -\phi_{0})}\\ G(0,0,0,4) & =\frac{\sin\;\alpha }{6a^{4}\;\cos^{3}\;\phi_{0}\;\cos^{3}(\alpha -\phi_{0})}[3\;\cos^{2}(\alpha -\phi_{0})+3\;\cos^{2}\;\phi_{0}-\sin^{2}\alpha ]\\ G(0,1,0,2) & =\frac{\sin\;\alpha }{2a^{2}\;\cos\;\phi_{0}\;\cos(\alpha -\phi_{0})}\frac{\cos\;\beta_{3}}{\cos\;\beta_{2}}\\ G(0,1,1,2) & =\frac{\sin\;\alpha }{2a^{2}\;\cos\;\phi_{0}\;\cos(\alpha -\phi_{0})}\frac{\cos\;\beta_{3}}{\cos\;\beta_{2}}\frac{2}{3}\left(1-\frac{\cos\;\beta_{3}}{\cos\;\beta_{2}}\right)\\ G(0,1,0,4) & =\frac{\sin\;\alpha }{6\;a^{4}\;\cos^{3}\;\phi_{0}\;\cos^{3}(\alpha -\phi_{0})}\frac{\cos\;\beta_{3}}{\cos\;\beta_{2}}\left[\cos^{2}(\alpha -\phi_{0})+2\;\cos^{2}\;\phi_{0}-\frac{\sin^{2}\alpha }{2}\right]\\ G(1,0,0,4) & =\frac{\sin\;\alpha }{a^{2}\;\cos\;\phi_{0}\;\cos(\alpha -\phi_{0})}\frac{\zeta_{23}}{6}\frac{\cos\;\beta_{3}}{\cos\;\beta_{2}}\\ G(1,1,0,4) & =\frac{\sin\;\alpha }{a^{2}\;\cos\;\phi_{0}\;\cos(\alpha -\phi_{0})}\frac{\zeta_{23}}{12}{\left(\frac{\cos\;\beta_{3}}{\cos\;\beta_{2}}\right)}^{2} \end{array}$$

$$\begin{array}{cc} G_{1,0}(0,0,0,3) & =\frac{\sin\;\alpha }{2a^{3}\;\cos\;\phi_{0}\;\cos(\alpha -\phi_{0})}\left[\frac{\cos\;\alpha }{\cos(\alpha -\phi_{0})}+\frac{1}{\cos\;\phi_{0}}\right]\\ G_{1,0}(0,0,0,5) & =\frac{\sin\;\alpha \;\cos\;\alpha }{3a^{5}\;\cos\;\phi_{0}\;\cos^{2}(\alpha -\phi_{0})}\\ & +\frac{\sin\;\alpha [\cos^{2}\;\phi_{0}+\cos^{2}(\alpha -\phi_{0})]}{12a^{5}\;\cos^{3}\;\phi_{0}\;\cos^{3}(\alpha -\phi_{0})}\left[\frac{3}{\cos\;\phi_{0}}+\cos\;\phi_{0}-3\frac{\sin(\alpha -\phi_{0})}{\cos(\alpha -\phi_{0})}\sin\;\phi_{0}\right] \end{array}$$

$$\begin{array}{cc} G_{1,0}(0,0,0,3) & =\frac{\sin\;\alpha }{6a^{3}\;\cos\;\phi_{0}\;\cos(\alpha -\phi_{0})}\frac{\cos\;\beta_{3}}{\cos\;\beta_{2}}\left[\frac{2\;\cos\;\alpha }{\cos(\alpha -\phi_{0})}+\frac{1}{\cos\;\phi_{0}}\right]\\ G_{1,0}(0,1,1,3) & =\frac{\sin\;\alpha }{6a^{3}\;\cos\;\phi_{0}\;\cos(\alpha -\phi_{0})}\frac{\cos\;\beta_{3}}{\cos\;\beta_{2}}\frac{1}{2}\left(1-\frac{\cos\;\beta_{3}}{\cos\;\beta_{2}}\right)\left[\frac{3\;\cos\;\alpha }{\cos(\alpha -\phi_{0})}+\frac{1}{\cos\;\phi_{0}}\right] \end{array}$$

G1,0(0,1,0,5)=sinα20a5cosϕ0cos(α−ϕ0)cosβ3cosβ2[4cosαcos(α−ϕ0)+2cosαcos3(α−ϕ0)]−[2sin(α−ϕ0)cos(α−ϕ0)(sinϕ0cos2(α−ϕ0)+sinϕ0cos2ϕ0)+1cosϕ0(3cos2(α−ϕ0)+1cos2ϕ0)]G1,0(1,0,0,5)=sinα2a3cosϕ0cos(α−ϕ0)[cosαcos(α−ϕ0)+1cosϕ0]ζ236cosβ3cosβ2G1,0(1,1,0,5)=sinα2a3cosϕ0cos(α−ϕ0)[3cosαcos(α−ϕ0)+2cosϕ0]ζ2330(cosβ3cosβ2)2

$$\begin{array}{cc} G_{0,1}(0,0,0,3) & =\frac{\sin^{2}\alpha }{2a^{3}\;\cos\;\phi_{0}\;\cos^{2}(\alpha -\phi_{0})}\\ G_{0,1}(0,0,0,5) & =\frac{\sin^{2}\alpha }{12a^{5}\;\cos\;\phi_{0}\;\cos^{2}(\alpha -\phi_{0})}\left[\frac{4}{\cos^{2}(\alpha -\phi_{0})}+\frac{2}{\cos^{2}\phi_{0}}-\frac{\sin^{2}\alpha }{\cos^{2}\phi_{0}\;\cos^{2}(\alpha -\phi_{0})}\right]\\ G_{0,1}(0,1,0,3) & =\frac{\sin^{2}\alpha }{3a^{3}\;\cos\;\phi_{0}\;\cos^{2}(\alpha -\phi_{0})}\frac{\cos\;\beta_{3}}{\cos\;\beta_{2}}\\ G_{0,1}(0,1,1,3) & =\frac{\sin^{2}\alpha }{4a^{3}\;\cos\;\phi_{0}\;\cos^{2}(\alpha -\phi_{0})}\frac{\cos\;\beta_{3}}{\cos\;\beta_{2}}\left(1-\frac{\cos\;\beta_{3}}{\cos\;\beta_{2}}\right)\\ G_{0,1}(0,1,0,5) & =\frac{\frac{\cos\;\beta_{3}}{\cos\;\beta_{2}}\sin^{2}\alpha }{4a^{5}\;\cos\;\phi_{0}\;\cos^{2}(\alpha -\phi_{0})}\left[\frac{1}{\cos^{2}(\alpha -\phi_{0})}+\frac{1∕3}{\cos^{2}\phi_{0}}-\frac{(\sin^{2}\alpha )∕5}{\cos^{2}\phi_{0}\;\cos^{2}(\alpha -\phi_{0})}\right]\\ G_{0,1}(1,0,0,5) & =\frac{\sin^{2}\alpha }{3a^{3}\;\cos\;\phi_{0}\;\cos^{2}(\alpha -\phi_{0})}\frac{\cos\;\beta_{3}}{\cos\;\beta_{2}}\frac{\zeta_{23}}{4}\\ G_{0,1}(1,1,0,5) & =\frac{\sin^{2}\alpha }{5a^{3}\;\cos\;\phi_{0}\;\cos^{2}(\alpha -\phi_{0})}{\left(\frac{\cos\;\beta_{3}}{\cos\;\beta_{2}}\right)}^{2}\frac{\zeta_{23}}{4} \end{array}$$

$$\begin{array}{cc} G_{2,0}(0,0,0,4) & =\frac{\sin\;\alpha }{6a^{4}\;\cos\;\phi_{0}\;\cos(\alpha -\phi_{0})}\left[\frac{2\;\cos^{2}\alpha +1}{\cos^{2}(\alpha -\phi_{0})}+\frac{3}{\cos^{2}\phi_{0}}-\frac{\sin^{2}\alpha }{\cos^{2}\phi_{0}\cos^{2}(\alpha -\phi_{0})}\right]\\ G_{2,0}(0,1,0,4) & =\frac{\sin\;\alpha (\cos\;\beta_{3}∕\cos\;\beta_{2})}{12a^{4}\;\cos\;\phi_{0}\;\cos(\alpha -\phi_{0})}\left[\frac{3\;\cos^{2}\alpha +1}{\cos^{2}(\alpha -\phi_{0})}+\frac{2}{\cos^{2}\phi_{0}}-\frac{\sin^{2}\alpha }{\cos^{2}\phi_{0}\cos^{2}(\alpha -\phi_{0})}\right]\\ G_{1,1}(0,0,0,4) & =\frac{\sin^{2}\;\alpha }{6a^{4}\;\cos\;\phi_{0}\;\cos^{2}(\alpha -\phi_{0})}\left[\frac{1}{\cos\;\phi_{0}}+\frac{2\;\cos\;\alpha }{\cos(\alpha -\phi_{0})}\right]\\ G_{1,1}(0,1,0,4) & =\frac{\sin^{2}\;\alpha }{12a^{4}\;\cos\;\phi_{0}\;\cos^{2}(\alpha -\phi_{0})}\left[\frac{1}{\cos\;\phi_{0}}+\frac{3\;\cos\;\alpha }{\cos(\alpha -\phi_{0})}\right]\frac{\cos\;\beta_{3}}{\cos\;\beta_{2}}\\ G_{0,2}(0,0,0,4) & =\frac{\sin^{3}\;\alpha }{3\;a^{4}\;\cos\;\phi_{0}\;\cos^{3}(\alpha -\phi_{0})}\\ G_{0,2}(0,1,0,4) & =\frac{\sin^{3}\;\alpha }{4\;a^{4}\;\cos\;\phi_{0}\;\cos^{3}(\alpha -\phi_{0})}\frac{\cos\;\beta_{3}}{\cos\;\beta_{2}}. \end{array}$$

The following are some identities we used to somewhat symmetrize cos *ϕ*_0_ and cos(*α* − *ϕ*_0_) in the integrals above.

(B.1) $$\frac{\cos\;\phi_{0}}{\cos(\alpha -\phi_{0})}=\frac{\tan\;\beta_{2}}{\tan\;\beta_{3}}\;\text{or}\;\cos\;\phi_{0}\frac{\sin\;\beta_{3}}{\sin\;\beta_{2}}=\cos(\alpha -\phi_{0})\frac{\cos\;\beta_{3}}{\cos\;\beta_{2}}.$$

## Appendix C. Equations containing higher order terms

For the total charge expression, a more complete expansion prior to the *ϕ* integral is given below

(C.1) q0=∫0αdϕ∫0β(ϕ)sinθdθσ(θ,ϕ)=∫0αdϕ{σ1(1−cosβ)+[(σ2−σ1)+λ(ϕ)(σ3−σ2)][C(1−cosβ)−DsinβC2+D2]}+{[D2(C2+D2)3∕2lnD+(C2+D2+C)tanβ2D−(C2+D2−C)tanβ2]}=σ1A+∫0αdϕ(σ2−σ1)+λ(ϕ)(σ3−σ2)3a2cos2(ϕ−ϕ0){1+10h~−3140a2cos2(ϕ−ϕ0)}+{252h~2−196h~+7391120a4cos4(ϕ−ϕ0)}+O(β9).

Both integrals (52) and (53) are expanded to higher orders prior to the *ϕ* integral. In the main text, we only keep the leading and the subleading terms for the *ϕ* integral.

(C.2) ∫0β(ϕ)sin2θdθ{σ1+sinθC(ϕ)sinθ+D(ϕ)cosθ[(σ2−σ1)+λ(ϕ)(σ3−σ2)]}=σ1(β2−sin(2β)4)+[(σ2−σ1)+λ(ϕ)(σ3−σ2)]{D(cos2β−1)−Csin2β4(C2+D2)}+{Cβ(C2+3D2)2(C2+D2)2−D3(C2+D2)2ln(cosβ+CDsinβ)}≈σ1(β2−sin(2β)4)+(σ2−σ1)+λ(ϕ)(σ3−σ2)4a3cos3(ϕ−ϕ0){−1+37−6h~30a2cos2(ϕ−ϕ0)}+{−140h~2+188h~−1131840a4cos4(ϕ−ϕ0)}+O(β10)

(C.3) ∫0β(ϕ)cosθsinθdθ{σ1+sinθC(ϕ)sinθ+D(ϕ)cosθ[(σ2−σ1)+λ(ϕ)(σ3−σ2)]}=σ1sin2β2+[(σ2−σ1)+λ(ϕ)(σ3−σ2)]{C(1−cos2β)−Dsin2β4(C2+D2)}−{Dβ(C2−D2)2(C2+D2)2+CD2(C2+D2)2ln(cosβ+CDsinβ)}≈σ1sin2β2+(σ2−σ1)+λ(ϕ)(σ3−σ2)3a2cos2(ϕ−ϕ0){1+10h~−4340a2cos2(ϕ−ϕ0)}+{28h~2−28h~+1391120a4cos4(ϕ−ϕ0)}+O(β9).
